# Structure-based design of stable recombinant hepatitis C virus E1E2 heterodimers

**DOI:** 10.1038/s41467-026-75744-9

**Published:** 2026-07-23

**Authors:** Joan Capella-Pujol, Fabian Mulder, Fabien Cannac, Stan Peters, Maddy L. Newby, Meliawati Poniman, Ian Zon, Wouter Olijhoek, Tim Beaumont, Max Crispin, Andrew B. Ward, Janke Schinkel, Rogier W. Sanders, Kwinten Sliepen

**Affiliations:** 1 Department of Medical Microbiology and Infection Prevention, Laboratory of Experimental Virology, Amsterdam UMC, University of Amsterdam, Amsterdam, the Netherlands; 2 Amsterdam institute for Immunology and Infectious diseases, Infectious diseases, Amsterdam, the Netherlands; 3 Department of Integrative Structural Biology and Computational Biology, The Scripps Research Institute, La Jolla, USA; 4 School of Biological Sciences, University of Southampton, Southampton, UK

**Keywords:** Protein vaccines, Protein vaccines, Hepatitis C virus

## Abstract

Hepatitis C virus (HCV) affects 47 million people and causes 239,000 deaths annually, yet no vaccine is on the horizon. Generating a stable native-like mimic of the envelope complex E1E2, the only target for known neutralizing antibodies, is an important aim for HCV vaccine development. Starting from a recombinant E1E2 design that utilizes a leucine zipper for proper folding, we used an iterative structure-based design approach to engineer antigens with at least 100-fold stronger binding to conformational antibodies and increased thermal stability. These new E1E2 designs facilitate production of native-like E1E2 antigens based on strains from different HCV genotypes and enable the generation of a recombinant E1E2 antigen design that lacks the immunogenic leucine zipper. A cryo-EM structure of one of the stabilized E1E2 antigens in complex with neutralizing antibody AT1211 provides atomic-level insights into an atypical epitope on the E2 subunit. Finally, immunogenicity studies in rabbits with adjuvanted E1E2 proteins show that immunogen stabilization alone does do not enhance serum neutralization breadth, but that removing the leucine zipper does increase homologous serum neutralization.

## Abstract (web summary)

The hepatitis C virus E1E2 glycoprotein is the only target of neutralizing antibodies. Here, the authors used structure-based design to generate soluble antigens that resemble the viral E1E2 heterodimer.

## Introduction

Hepatitis C virus (HCV) continues to be a global health burden with an estimated 47 million people living with chronic HCV infection^1^. Despite the high efficacy of direct-acting antivirals (DAAs), which can achieve cure rates up to 95%, several factors hamper HCV eradication programs. First, because initial infection causes few or no symptoms, most infected individuals are unaware of their infection. Second, DAA treatment does not provide protection against reinfection and, third, most low-income countries and underprivileged populations in high income countries have limited access to DAAs. As a result, HCV causes around 1 million new infections and ~239,000 deaths globally each year^1^. A vaccine that effectively prevents HCV (re)infection is crucial to overcoming these challenges and remains a critical unmet need^2,3^.

HCV is an enveloped positive-sense, single-stranded RNA virus that belongs to the *Flaviviridae* family. Its genome encodes nonstructural proteins, which are responsible for mediating viral processing inside the host cell, as well as E1 and E2, the envelope membrane-bound glycoproteins that form heterodimers, and possibly homodimers of heterodimers^4^, and are involved in viral entry. E2 serves as the receptor binding-subunit, interacting with scavenger-receptor class B1 (SR-B1) and tetraspanin CD81 on the host cell to facilitate entry^5–7^. Although the precise mechanism by which E1E2 mediates membrane fusion and viral entry remains unclear, E1E2 is thought to undergo conformational changes after E2 binds to SR-B1 followed by binding to CD81. It is thought that the E1 subunit facilitates membrane fusion by engaging its putative fusion peptide (pFP), possibly triggered by low pH-induced conformational changes^8–10^.

E1E2 is the primary target for neutralizing antibodies (NAbs) and therefore important for the development of an effective HCV vaccine. However, the high genetic diversity, heavy glycosylation, and conformational heterogeneity of E1E2 have posed significant challenges for an E1E2-based vaccine^11–13^. The recently solved structures of the E1E2 heterodimer in membrane-bound and soluble forms provide a foundation for structure-based E1E2 vaccine design^4,14,15^. The known three classes of viral fusion protein contain a metastable active prefusion form that can be triggered, often by the target cell receptor, to undergo conformational changes to the low-energy postfusion form to initiate viral entry^16^. Stabilization of the prefusion form is key for most viral vaccines design as this conformation presents all relevant neutralizing epitopes^16–20^. Since E1E2 possibly represents a novel fourth class of viral fusion protein^21^ and its precise fusion mechanism still needs to be elucidated, it is not yet completely clear if and which of the published E1E2 structures resemble the presumed prefusion state. Still, a key objective of HCV vaccine design is to stabilize E1E2 in a native-like conformation, which optimally presents key conformational neutralizing epitopes and thus mimicking the antigenicity of infectious E1E2 on the virus.

Recently, Guest and colleagues described a secreted soluble version of E1E2 (sE1E2), in which both the E1 and E2 transmembrane domains were replaced with human-derived Jun/Fos leucine zipper (LZ) and the natural protease site was substituted with an engineered furin site (sE1E2.v1-LZ)^22^. This sE1E2.v1-LZ is thought to represent a native-like mimic of E1E2, because it binds the broadly neutralizing antibody (bNAb) AR4A, which usually only recognizes native membrane-associated E1E2^14,23^. However, while the immunogenicity of sE1E2.v1-LZ was somewhat improved compared to sE2 alone, the LZ domains induced strong antibody responses^24^. These antibodies are possibly autoreactive in humans, suggesting that sE1E2.v1-LZ may not be suitable for human vaccination^24^. Therefore, we aimed to design improved recombinant E1E2 antigens for use in (pre-)clinical vaccine studies.

The emergence of artificial intelligence (AI) tools such as AlphaFold offers new opportunities for protein engineering and the design of E1E2 antigens^25,26^. Furthermore, extensive mutagenesis studies have identified regions and amino acids crucial for E1E2 folding, infectivity and antigenicity^8,27^. Here, we combined rational design based on high-resolution structures and computational predictions and data from previously published alanine screening studies to iteratively engineer novel sE1E2 antigens, including ones that do not require the use of heterologous dimerization domains, such as Jun/Fos, SYNZIP for proper folding. These designs resulted in native-like E1E2 antigens from different HCV strains and genotypes, displaying enhanced antigenicity and greater thermostability than previous designs. Immunization studies in rabbits demonstrated that the constructs lacking heterodimerization domains induced a more E1E2-focused immune response. A cryo-electron microscopy (cryo-EM) structure of E1E2 from genotype 1a AMS0232 strain in complex with antibody AT1211 revealed an atypical neutralization epitope involving interactions with the framework region 3 (FR3). The rational design work presented here provides a foundation for the next-generation of E1E2 immunogens.

## Results

### Replacing the putative fusion peptide by a hydrophilic linker improves native-like folding of soluble E1E2

**Fig. 1:**
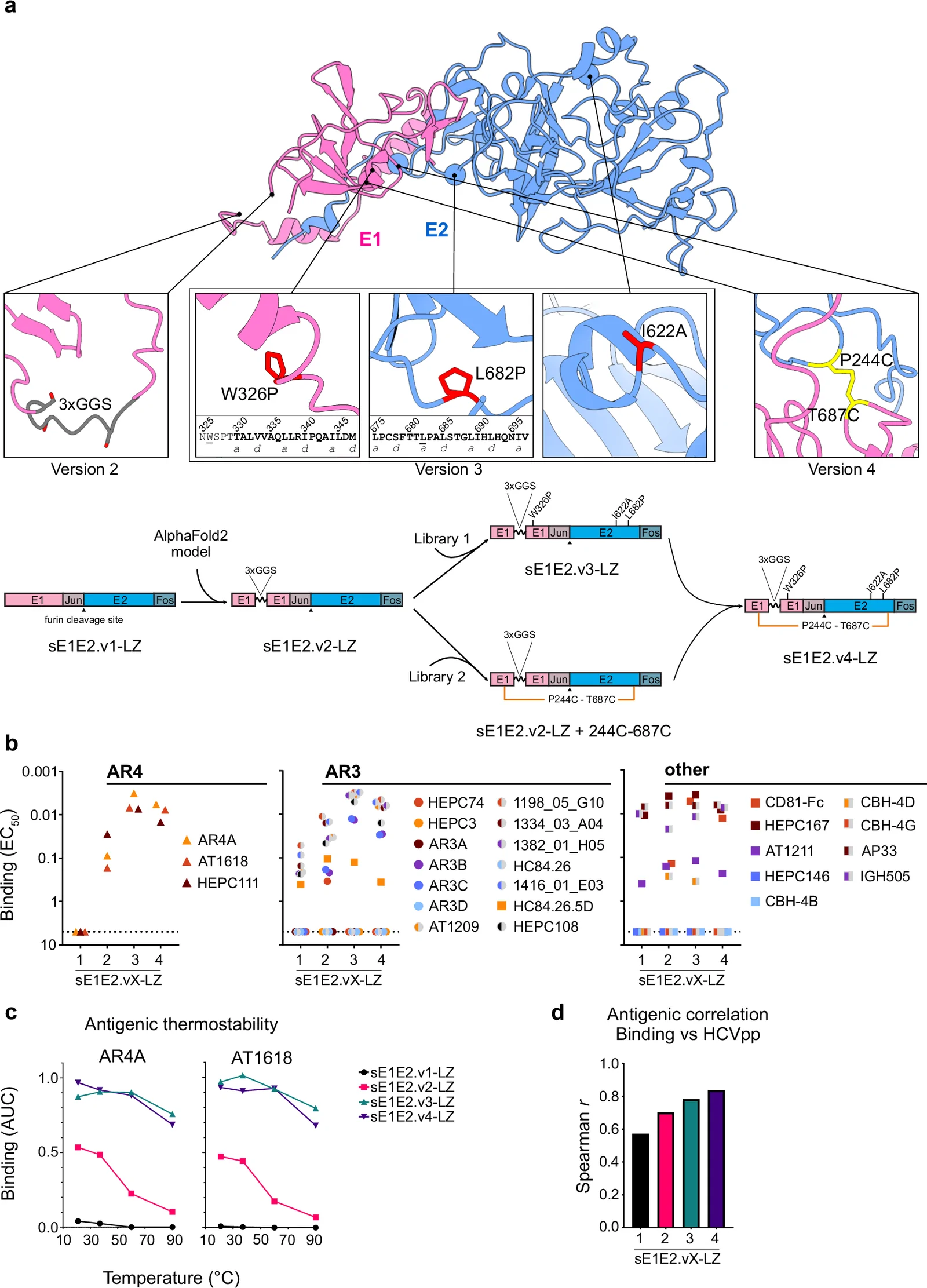
Design of a hyperstable soluble E1E2. **a** Predicted model representation of sE1E2.v4-LZ. Mutations are depicted and indicated on the cryo-EM structure of the E1E2 complex (PDB: 7T6X). AlphaFold2 (AF2) Multimer predictions of the stabilized versions were run and the predicted mutants are depicted in the squares. The putative heptad repeat region sequences of AMS0232 E1 and E2 are depicted in the W326P and L682P panels with the putative heptad region in bold, 326 and 682 underlined, and the heptad *a* and *d* positions below in italics. The schematic below visualizes the design campaign with details on the AMS0232 sE1E2 versions. **b** Binding data obtained by ELISA from 25 antibodies and CD81-Fc tested against the different sE1E2 summarized in three dot plots, grouping AR4-directed bNAbs (left plot); AR3-directed bNAbs (middle); and others (right). ELISA EC_50_ values from experiments performed in duplicate are plotted on the *y* axis (see Figure S2e for the individual binding curves). **c** Antigenic thermostability. ELISA results showing residual binding of conformational antibodies AR4A and AT1618 (normalized to linear antibody AP33 binding) to all constructs after being heated at different temperatures for one hour. All experiments were performed in duplicate. **d** Spearman correlation values (*r*) for antibody binding to sE1E2.v4-LZ from (**b**) *versus* neutralization of the matched AMS0232 HCVpp, plotted as bargraphs. The correlation plots can be found in Figure S3b. Source data are provided as a Source Data file.

We used the genotype 1a strain AMS0232 E1E2 sequence (Genbank: OL855837.1) as starting point for our structure-based design campaign. This neutralization-resistant strain was obtained from an HCV-infected individual enrolled in the MOSAIC cohort and it was used to solve the first high-resolution cryo-EM structure of E1E2, which was complexed with bNAb AR4A (PDB: 7T6X)^14,28^. This structure contains several structurally unresolved regions. We used AlphaFold2 to predict the structures of E1 and E2 subunits to complement the experimental E1E2 structure^9,14^. Overlaying the AlphaFold2 E1 prediction with the experimental E1 structure suggested that the hydrophobic helix-loop-helix of the pFP is (partly) buried within the membrane^9,14^ (Fig. S1b). Additionally, analyses using a number of prediction tools implied that the pFP constitutes a transmembrane domain^29–32^ (Fig. S1c). Finally, the structure of the membrane-bound E1E2 homodimer was published after our predictions and analyses were performed, and it confirmed that the pFP is inserted in the membrane^4^. We hypothesized that the hydrophobic membrane-associated pFP (residues 276-286)^33^ might hamper proper folding and efficient expression of a soluble recombinant E1E2 construct. To test this, we first generated a version 1 (v1) soluble E1E2 based on the AMS0232 sequence using a previously described recombinant E1E2 design (sE1E2.v1-LZ)^22^, that consists of the complete E1 and E2 ectodomains fused to a human-derived Jun/Fos leucine zipper (LZ) to facilitate E1/E2 heterodimerization^22^ (Fig. 1a, and Fig. S1a). Next, we replaced the pFP (residues 261-294) in sE1E2.v1-LZ with a short hydrophilic 9 amino acid flexible linker (GGSGGSGGS), which we refer to as sE1E2.v2-LZ (Fig. 1a, and Fig. S1a).

Both sE1E2.v1-LZ and sE1E2.v2-LZ yielded similar protein quantities following Strep-TactinXT affinity purification from the supernatant of transfected HEK 293F cells. However, size exclusion chromatography (SEC) and subsequent non-reducing SDS-PAGE revealed that sE1E2.v1-LZ produced more higher molecular weight species and undesired disulfide-linked E1E2 species (Fig. S1d, e). The pFP contains two cysteines that may contribute to the disulfide scrambling observed in sE1E2.v1-LZ, while their removal yielded a more homogeneous E1E2 protein.

HCV pseudoparticles (HCVpp) in which the pFP was replaced by the same 9-amino acid linker did contain E1E2 but failed to infect otherwise susceptible Huh-7 cells. This confirms that while E1E2 lacking the pFP can be incorporated into HCVpp, the pFP is crucial for infection (Fig. S1f).

Next, we compared the antigenic structures of sE1E2.v1-LZ and sE1E2.v2-LZ using ELISA (Fig. S1g). Overall, sE1E2.v2-LZ exhibited enhanced binding to conformation-sensitive bNAbs. BNAbs that target the metastable antigenic region 4 (AR4) and that are dependent on the correct conformation of the E1E2 heterodimer, such as AR4A and AT1618, showed overall increased binding indicated by the higher maximal binding signal  and ~ 4-fold and ~3-fold lower half-maximal effective binding concentrations (EC_50_), respectively (Fig. S1g). Furthermore, sE1E2.v2-LZ also gained binding to AR3C and HEPC74, bNAbs targeting antigenic region 3 (AR3), and to the Fc-tagged receptor mimic of CD81 (CD81-Fc) (Fig. S1g). In summary, replacing the pFP with a short linker improves the antigenicity of recombinant soluble E1E2 and is in line with what was reported recently by another group^34^.

### Iterative structure-based design enhances antigenicity of sE1E2.v2-LZ

Next, we used structure-guided design to construct two mutational libraries to further enhance the antigenicity and stability of sE1E2.v2-LZ. We mostly mutated conserved amino acids in the core of the E1E2 heterodimer in order to improve overall folding, while not directly affecting bNAb epitopes. The first mutational library contained over thirty individual amino acids substitutions, including proline residues to stabilize loops, large hydrophobic residues to improve packing, and alanine residues that were previously identified in an alanine-scanning study on membrane-bound E1E2^8,35^. (Fig. 1a and Fig. S2a, left panel). The second mutational library contained nine cysteine mutations in the core of E1 and E2, aimed to covalently link E1 and E2 via disulfide bonds (Fig. 1a and Fig. S2a, right panel).

Both libraries were transfected in HEK 293T cells, and unpurified supernatants were screened against a small panel of antibodies and Fc-tagged CD81 (CD81-Fc). In the first library, W326P and L682P substitutions markedly improved the AR4A and AT1618 binding signal (Fig. S2b, and top panel). Interestingly, W326P and L682P are located upstream or at the *a* position of the putative heptad repeats of E1 and E2, respectively (Fig. 1a). Disruption of these heptad repeats by introducing prolines in their *a* and *d* positions is known to impede HCV entry^33,36^. Additionally, I622A increased binding to CD81-Fc and AR3C (Fig. S2b, and top panel). We combined W326P, L682P and I622A, which further augmented AR4A and AT1618 binding compared to sE1E2.v2-LZ and the single mutants. We designated this construct sE1E2.v3-LZ (Fig. 1a, and Fig. S2a).

In the second library, four out of five introduced double-cysteine substitutions strongly decreased or abolished AR4A and AT1618 binding in sE1E2.v2-LZ (Fig. S2b, bottom panel). Only P244C-T687C retained AR4A and AT1618 binding (Fig. S2b, bottom panel), while the single P244C or T687C substitutions abolished binding. This suggested that 244C in E1 and 687C in E2 form an intermolecular disulfide bond. We incorporated P244C and T687C in sE1E2.v3-LZ to generate sE1E2.v4-LZ (Fig. 1a, and Fig. S2a).

Next, we purified the four sE1E2 versions from HEK 293F using Strep-TactinXT chromatography. While the peak representing the E1E2 monomer reached ˜80% height of the peak representing dimers and aggregates for sE1E2.v1-LZ (Fig. S1d), the ratio was reversed for sE1E2.v2-v4-LZ indicating that the latter designs produced relatively more monomers (Fig. S1d and Fig. S2c). In particular, sE1E2.v4-LZ consistently revealed a slightly narrower elution peak for the heterodimer (Fig. S2c). All proteins were efficiently cleaved by furin (Fig. S2d). Importantly, non-reducing SDS-PAGE gels revealed a single band around 130 kDa for sE1E2.v4-LZ, indicating the presence of the designed 244C-687C intermolecular disulfide bond between E1 and E2 (Fig. S2d).

We then probed the purified proteins using a panel of 25 HCV monoclonal antibodies (mAbs) targeting known neutralizing and non-neutralizing epitopes, along with a CD81-Fc construct in ELISA^37–43^. Both sE1E2.v3-LZ and sE1E2.v4-LZ showed markedly enhanced binding to most tested conformational bNAbs compared to sE1E2.v1-LZ (Fig. 1b, and Fig. S2e). Conformational E1E2-dependent bNAbs AR4A, AT1618 and HEPC111 displayed 800-, 600- and 300-fold lower EC_50_ values compared to sE1E2.v1-LZ and at least 15-, 20- and 2-fold lower compared to sE1E2.v2-LZ, respectively (Fig. 1b, and Fig. S2d). We also observed higher maximum binding plateaus for the ELISA curves for these bNAbs, indicating that more sE1E2.v2-v4-LZ molecules in the ELISA wells present these epitopes than sE1E2.v1-LZ (Fig. S2e). Additionally, sE1E2.v3-LZ and sE1E2.v4-LZ also displayed stronger binding to AR3 bNAbs (Fig. 1b, and Fig. S2e), such as AR3C (390- and 170-fold compared to sE1E2.v1-LZ), AT1209 (59- and 32-fold) and HEPC108^41,44^ (45- and 15-fold) and of CD81-Fc (900- and 400-fold). Importantly, non-neutralizing mAbs (non-Nabs) CBH-4B, CBH-4D and CBH-4G showed reduced binding to sE1E2.v4-LZ.

### sE1E2.v3-LZ and sE1E2.v4-LZ are thermostable antigenic mimics of virion-associated E1E2

To compare antigenic stability, we performed thermostability ELISA assays. We incubated sE1E2.v1-v4-LZ at 21 °C, 37 °C, 60 °C, and 90 °C for one hour and tested residual binding to conformation-sensitive bNAbs AR4A and AT1618, as well as the non-conformational bNAb AP33. AP33 binding was not affected by temperature change, as expected, and was used to normalize the binding of the conformational antibodies (Fig. 1c). After incubation at 60 °C and 90 °C, sE1E2.v1-LZ and sE1E2.v2-LZ lost all or most of the detectable binding to AR4A and AT1618. The 60 °C and 90 °C incubations barely affected the binding of sE1E2.v3-LZ and sE1E2.v4-LZ to AR4A and AT1618, suggesting that these conformational epitopes were remarkably thermostable on sE1E2.v3-LZ and sE1E2.v4-LZ, but not on sE1E2.v1-LZ and sE1E2.v2-LZ (Fig. 1c). Subjecting all proteins to freeze-thaw stress by applying up to six freeze-thaw cycles did not significantly affect their antigenicity (Fig. S3a).

To further assess whether the recombinant sE1E2 constructs are antigenic mimics of virion-associated E1E2, we plotted the ELISA binding EC_50_ values against the neutralization midpoint inhibitory concentration (IC_50_) values of the sequence-matched AMS0232 HCVpp (Fig. 1d, and Fig. S3b). The neutralization IC_50_ values of AMS0232 HCVpp correlated strongly and significantly with the binding EC_50_ values of AMS0232 sE1E2.v3-LZ and sE1E2.v4-LZ (Spearman *r* = 0.78; *p* < 0.0001 and *r* = 0.84; *p* < 0.0001, respectively), while correlation was weaker for sE1E2.v2-LZ (*r* = 0.70: *p* < 0.0001) and weakest for sE1E2.v1-LZ (*r* = 0.57; *p* = 0.001) (Fig. 1d, and Fig. S3b). In summary, sE1E2.v3-LZ and sE1E2.v4-LZ display enhanced binding to conformational bNAbs and are close antigenic mimics of infectious E1E2 on the virion, while E1E2.v4-LZ also displayed reduced binding to non-NAbs.

### Structural characterization of sE1E2.v4-LZ in complex with NAb AT1211

**Fig. 2:**
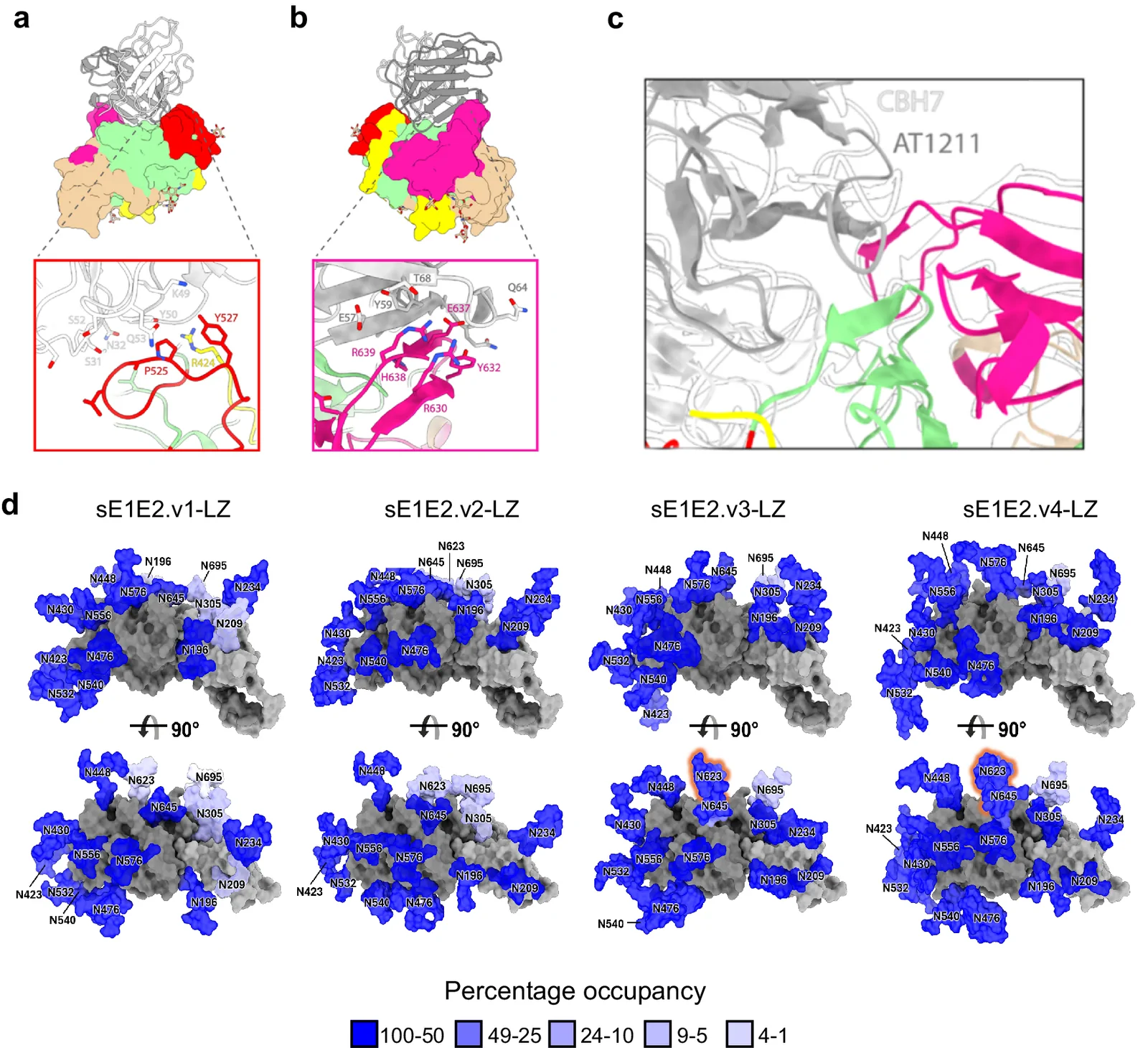
Cryo-EM structure of sE1E2.v4-LZ in complex with NAb AT1211 and glycan occupancy models. **a**, **b** The newly solved AT1211 epitope. In (**a**) and (**b**), the β-sandwich domain is shown in green, the CD81 apical binding site in red, the back layer in magenta, the front layer in yellow and the VR region 3 and 4 in tan. AT1211 LC CDR loops–CD81 binding site interactions are shown in (**a**), while (**b**) reveals AT1211 HC framework 3–β hairpin interactions. **c** Comparison of AT1211 and CBH7 mAbs binding to E2. AT1211 and E2 are colored as previously, CBH7 is white, outlined in black. **d** Glycan occupancy analysis. Glycans were modeled onto structure 7T6X using GlycoShape^113^ and glycan occupancy is represented in blue, with low occupancy in light blue and high occupancy in darker blue. Detailed results on site-specific resolution are found in Fig. S11.

We then proceeded to structurally characterize the newly generated sE1E2.v4-LZ construct by complexing it with the moderately neutralizing antibody (NAb) AT1211 (Fig. S5), of which the epitope is designated as domain C and known to involve W549 and R639 but was not yet structurally resolved^39^. We obtained a 3.0 Å resolution structure of sE1E2.v4-LZ in complex with the antibody fragment antigen-binding region (Fab) of AT1211 using cryo-EM (Fig. 2a–c, Fig. S4, Table S1), revealing a new HCV NAb epitope at atomic detail. The antigen structure itself is similar to the membrane-bound AMS0232 E1E2 heterodimeric complex (7T6X^14^), with an RMSD of 0.853 Å across 187 pruned pairs of residues, and 1.291 Å across all 203 common pairs of modeled residues, measured on their Cα atom. The structure did not reveal the entire E1E2 heterodimer. While complexing and imaging with other antibodies such as IGH505 (binding E1^14^) showed a clear, multi-Fab complex indicating that the whole heterodimer is present in our sample, the flexibility of the complex hindered our ability to solve a full-length structure (Fig. S6).

Nevertheless, the structure revealed the complete epitope of AT1211. This NAb utilizes an extensive paratope region totaling a buried surface area (B.S.A.) of 1244 Å^2^ and its epitope is located opposite of the glycan shield of E2 (Fig. 2a, b), targeting the β-sandwich domain, the β-hairpin of the E2’s back layer and bordering the CD81 binding site^14^. The paratope can be divided between a first, conventional interface mediated by AT1211’s complementarity-determining region (CDR) loops from its heavy chain (HC) and light chain (LC), and a second adjacent interface mediated by the framework 3 (FR3) region on its HC. The CDR loops of AT1211 bind through a rich network of electrostatic and van der Waals interactions, mostly focused on the β-sandwich and CD81 binding domain. The LCDR2 generates several electrostatic contacts and hydrogen bonds with the neighboring E2’s R424, P525 and Y527 from the back layer and CD81 binding domains (Fig. 2b), whereas the LCDR1 and LCDR3 domains interact mainly with the backbone of E2 β-sandwich through hydrogen bonds. Because of the interaction with AT1211, the CD81 binding loop is locked in its retracted form, described elsewhere^10^ (Fig. S7a). Furthermore, when superimposed with the structure of E2 bound to the CD81 large extracellular loop (CD81-LEL; PDB: 7MWX)^1^ the HC of AT1211 directly clashes with residues 78-82 of the CD81-LEL, possibly impeding proper CD81 binding (Fig. S7b). This same region is also heavily coordinated with residues on the HCDR3 (D96, T100A and P100B) while Y97, F98 and W99 interact with hydrophobic amino acids on the β-strands formed by E2’s residues 507-515, sandwiched on the other side by the HCDR2 domain of AT1211. The HCDR1 region is not involved in any interaction with the E2 antigen. Supplementing the CDR interactions, AT1211 utilizes its FR3 strands to interact with a second region consisting of the β-hairpin of E2 at position 627 (Fig. 2b). The β-strands align on a plane in a continuous antiparallel β-sheet between the framework of AT1211 and the E2, with a dense network of hydrogen bonds between both domains. This is also reflected in the buried surface area between the antibody chains and E2 protein, with a B.S.A. of 840 Å^2^ for the HC - E2 interaction, nearly double the surface area of the LC - E2 interface at 493 Å^2^. The unusual FR3 – E2 contact area alone accounts for 415 Å^2^, thus contributing about half of the HC interactions.

We inspected the residues involved in the interaction and compared them to the IGHV1-69*18 germline sequence of AT1211 (Fig. S8) and observed that both the HCDR2 and HFR3 regions have undergone somatic hypermutation, presumably to improve binding to E2. Notably, a pair of isoleucine and leucine residues (I53, L83) is mutated to shorter valine and serine residues, respectively, most likely to avoid clashes, while A57 is mutated to a glutamate residue that facilitates an interaction with R639 on the viral protein.

We compared the structure of AT1211 with the recently published^45^ structure of NAb CBH7, which binds to a similar region (domain C) on E2 and with a similar approach (PDB: 8THZ). AT1211’s HC binds significantly closer to the β-sandwich (Fig. 2c). Furthermore, CBH7 displays several bulkier or longer residues than AT1211, resulting in an upward shift of the HCDR2, notably at positions 50, 54 and 58 (T/S/T in AT1211, and E/F/K in CBH7; Fig. S9).

### Stabilized recombinant E1E2 have a higher glycan occupancy

Viral *N*-linked glycans play a crucial role in the E1E2 folding, particularly glycans at positions N196 and N305 within the E1/E2 interface^14^. We observed densities in the cryo-EM map belonging to glycans on positions N556, N623, and N645 on E2 (Fig. S10). To provide a more complete picture of the glycan shield, its composition on sE1E2.v4-LZ and on our other stabilized immunogen designs, we performed site-specific glycan analysis covering all potential glycosylation sites (PNGS) (Fig. S11a, S14d). All versions showed enrichment for complex-type glycans compared to oligomannose-type. However, we observed some major differences across the four sE1E2 versions. PNGS N209 and N305 in E1, and N623 and N645 in E2, were poorly glycosylated ( < 50% occupancy) on sE1E2.v1-LZ (Fig. 2d). Although *N-*linked glycans are usually located at NXT or NXS sequons (X, any amino acid except for proline), E2 contains a noncanonical NXV motif at N695 which is partially occupied^14^. Glycan occupancy at this site was low for each construct, with maximum occupancy of ~20% for sE1E2.v3-LZ (Fig. 2d). Similarly, compared to sE1E2.v3-4-LZ we also observed low occupancy at N305, N623, and N645 (22%, 11% and 63% occupancy, respectively) for sE1E2.v2-LZ, while N209 occupancy was improved (Fig. S11c).

We hypothesized that the I622A mutation in sE1E2.v3-LZ and in sE1E2.v4 located at the −1 position of the N623 PNGS might affect occupancy at N623. Indeed, site-specific glycan analysis on purified sE1E2.v2-LZ with the I622A mutation confirmed that this mutation increases glycosylation at N623 (Fig. S11). The observation that an alanine at the −1 position is more advantageous than isoleucine is consistent with previous studies on the amino acid bias in positions surrounding the PNGS^46,47^. The overall higher glycan occupancy of sE1E2.v3-LZ and sE1E2.v4-LZ more closely resembles that of membrane-associated full-length E1E2 and this might have contributed to their more desirable antigenicity profile. For example, the glycan on 623 interacts with AR4A and increased glycosylation might therefore enhance binding to this bNAb, while enhanced glycosylation of N305 might improve the structural integrity of the E1E2 heterodimer complex^14^ and thus enhance AR4A and AT1618 binding^23,48^.

### Generating native-like E1E2 heterodimers from different strains

To determine whether the recombinant HCV E1E2 designs are generally applicable across HCV strains, we generated all engineered versions of heterodimers from four different strains: H77, the prototypic genotype 1a strain that was used to produce the original sE1E2.v1-LZ heterodimer^22^; AMS0230, a neutralization-resistant genotype 1a strain^43^; AMS3a, a genotype 3 strain isolated from a chronically infected individual^49^; and UKNP4.1.1, a genotype 4 strain that engages several inferred germline antibodies^50^. All sixteen constructs yielded E1E2 proteins that were fully cleaved (Fig. S12a). In agreement with previous observations with the AMS0232 strain, the new four sE1E2.v1-LZ constructs produced more disulfide-scrambled high molecular weight species than the more stabilized sE1E2.v2-v4-LZ constructs (Fig. S12a). Notably, the four SEC-purified sE1E2.v4-LZ proteins all migrated as a single band on non-reducing SDS-PAGE, indicating that the 244C-687C disulfide bond was formed efficiently.

**Fig. 3:**
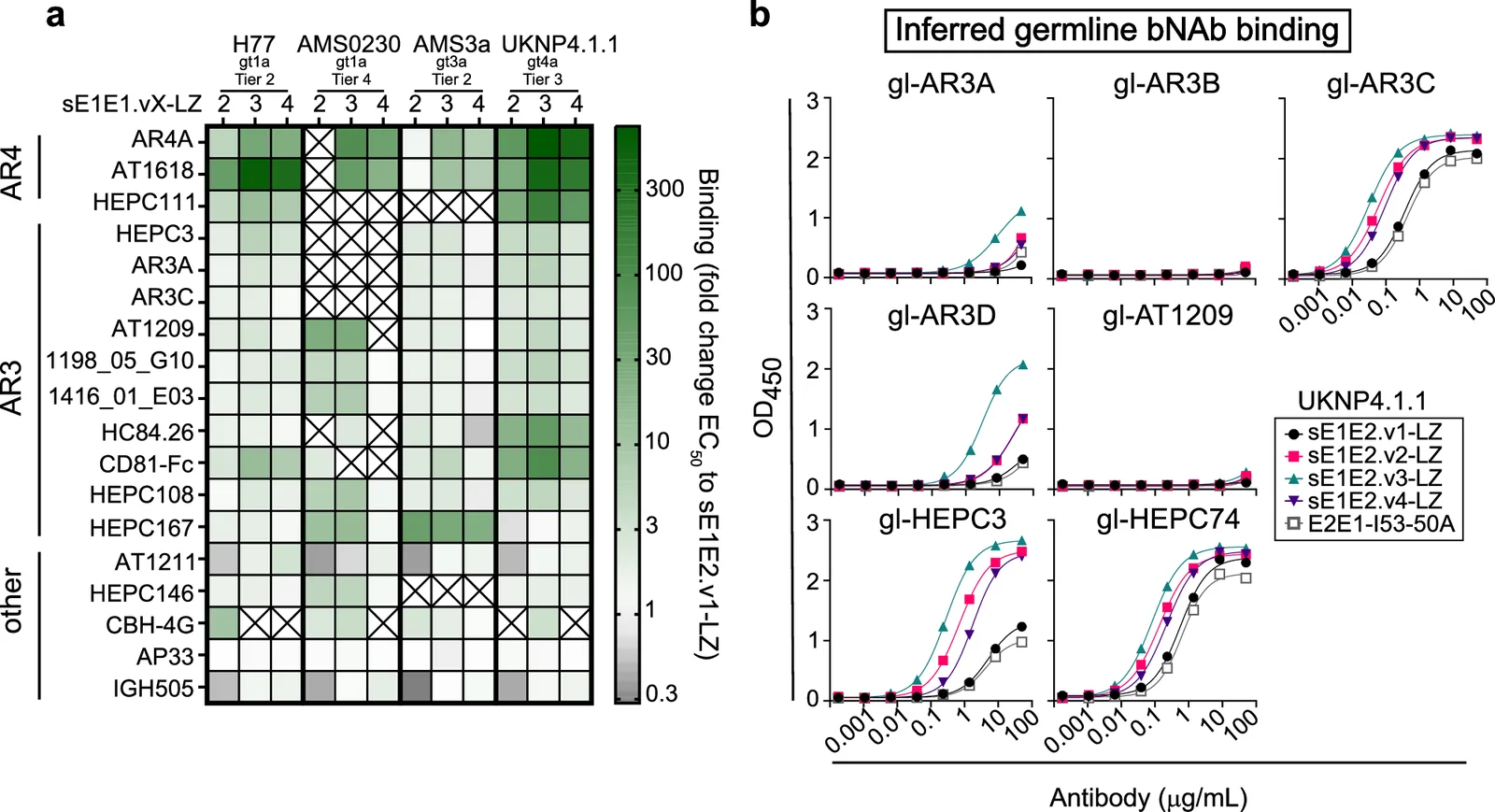
Stabilizing mutations are broadly applicable to other strains. **a** The set of mutations from versions 2 to 4 were applied to strains H77 (genotype 1a), AMS0230 (genotype 1a), AMS3a (genotype 3a) and UKNP4.1.1 (genotype 4a) and tested against a reduced panel of 17 bNAbs and CD81-Fc. The EC_50_ for each bNAb was calculated from duplicate experiments (data from Fig. S12b). The logarithm of the fold change in EC_50_ (sE1E2.v1-LZ/sE1E2.vX-LZ) is depicted and summarized for all results in a heatmap. Green represents an increase in binding (i.e., lower EC_50_), white shows no change in binding, while gray represents less binding than version 1. Crossed cells indicate no detectable binding at the maximum concentration tested (5.0 μg/mL). The tier categorization for neutralization sensitivity of the viruses is shown in the top of the heatmap as determined by Chumbe and colleagues^43^ (**b**) Comparison of UKNP4.1.1 sE1E2.v1-v4 to previously studied UKNP4.1.1 E2E1-I53-50A^50^. The same panel of inferred germline AR3C-class bNAbs was tested in ELISA and full ELISA curves are shown. Each dot represents the mean from two technical replicates. Source data are provided as a Source Data file.

We compared the antigenic profiles of the sixteen recombinant E1E2 designs using ELISA with a panel of seventeen mAbs and CD81-Fc. As observed for AMS0232, the antigenic properties of sE1E2.v3-LZ and sE1E2.v4-LZ were superior to that of sE1E2.v1-LZ (Fig. 3a). Specifically, AR4A and AT1618 binding improved significantly indicating that the modifications improve native-like folding of recombinant sE1E2: the EC_50_ values for AR4A and AT1618 were improved at least 7-fold and up to 700-fold for AMS3a, H77 and UKNP4.1.1 sE1E2.v3-LZ and sE1E2.v4-LZ when compared to their sE1E2.v1-LZ counterparts. The AMS0230 viral strain is unusually neutralization-resistant and only the AR4A and AT1618 bNAbs are known to neutralize it^43^. Interestingly, AR4A and AT1618 did not detectably bind AMS0230 sE1E2.v1-LZ nor sE1E2.v2-LZ in ELISA, while both bNAbs were able to bind AMS0230 sE1E2.v3-LZ and sE1E2.v4-LZ. Furthermore, for mAbs that do not neutralize AMS0230, we observed no or weak binding to sE1E2.v4-LZ, indicating that this design closely recapitulates the antigenicity of this neutralization-resistant strain.

Finally, most AR3-targeting bNAbs displayed improved binding to sE1E2.v2-LZ compared to sE1E2.v1-LZ, but this did not improve further when adding the stabilizing mutations from sE1E2.v3-LZ or sE1E2.v4-LZ (Fig. 3a, and Fig. S12b). In summary, the engineered sE1E2 constructs serve as effective design templates that are applicable to a variety of different HCV strains and generate native-like recombinant E1E2 heterodimers with a desirable antigenic profile.

### Stabilizing mutations enhance binding to AR3 bNAb precursors

To induce bNAbs, it is necessary to engage the cognate B cell germline precursors that have the capacity to develop such bNAbs. Germline-targeting vaccination aims to recapitulate this process by priming with immunogens that are specifically engineered to target bNAb precursor B cells. Results from recent phase I clinical trials have shown that germline-targeting is arguably the most promising strategy to induce human immunodeficiency virus 1 (HIV-1) bNAbs by vaccination^51,52^. For HCV, most immunogens and viral strains react to none or only a few of the known precursor bNAbs. Therefore, it is unlikely that these immunogens would engage the cognate precursor B cells and are thus also unlikely to consistently induce bNAbs^50,53^. We previously found that HCV glycoproteins based on the UKNP4.1.1 strain engage a number of inferred precursor bNAbs and their B cell counterparts in vitro^50^. However, this HCV glycoprotein design (E2E1-I53-50A) displays non-native-like antigenicity, as it does not bind AR4A and AT1618^50^. Therefore, we used this strain and design to benchmark the stabilized heterodimer designs using the same panel of inferred germline bNAbs (gl-bNAb) in ELISA tested in our previous study (Fig. 3b)^49,50^. Applying the sE1E2.v1-LZ design to UKNP4.1.1 sequence did not notably improve inferred germline bNAb binding compared to the non-native E2E1-I53-50A design (Fig. 3b). In contrast, applying the sE1E2.v2-LZ design to UKNP4.1.1 sequence resulted in 4- to 7-fold stronger binding of gl-AR3C, gl-HEPC3 and gl-HEPC74 compared to UNKP4.1.1 E2E1-I53-50 and sE1E2.v1-LZ, and their binding was further enhanced, albeit subtly, when sE1E2.v3-LZ was applied. Additionally, incorporating the sE1E2.v3-LZ enabled binding to gl-AR3D. Adding the disulfide bond from sE1E2.v4-LZ decreased the binding signal compared to sE1E2.v3-LZ and remained similar to that of sE1E2.v2-LZ.

### Generation of soluble native-like E1E2 (C2P2) devoid of a heterologous dimerization domain

The LZ used here is a human-derived dimerization domain, which complicates application in humans^22^, because of the risk of inducing auto-antibodies. Therefore, we tested whether the E1E2.v2-v4 designs would enable fusion to another, non-human dimerization domain. We fused the synthetic coiled-coil SYNZIP (SZ) to the E1 and E2 C-termini instead of the LZ (i.e., Jun/Fos domains) (sE1E2.v2-v4-SZ, Fig. S13a)^15,54^. We purified these constructs as before, and we observed no significant impact of replacing LZ by SZ on protein yields, furin cleavage or the formation of the 244C-687C disulfide bond (Fig. S13b). Testing the same panel of seventeen mAbs and CD81-Fc revealed that the domain exchange had a negligible effect on antigenicity (Fig. S13c) as the EC_50_ values of LZ- and SZ-derived sE1E2.v2-v4 proteins were strongly correlated (*p* < 0.0001; Spearman *r* = 0.92) (Fig. S13d). We conclude that the sE1E2.v2-v4 designs are compatible with the SZ heterodimerization domains.

LZ or SZ are essential for heterodimerization and native-like folding of recombinant previous sE1E2 designs^22^. However, as heterologous oligomerization domains are known to be immunogenic and can potentially interfere with the induction of desired on-target (N)Ab responses^24,55,56^, it would be preferred to dispense with such domains altogether.

**Fig. 4:**
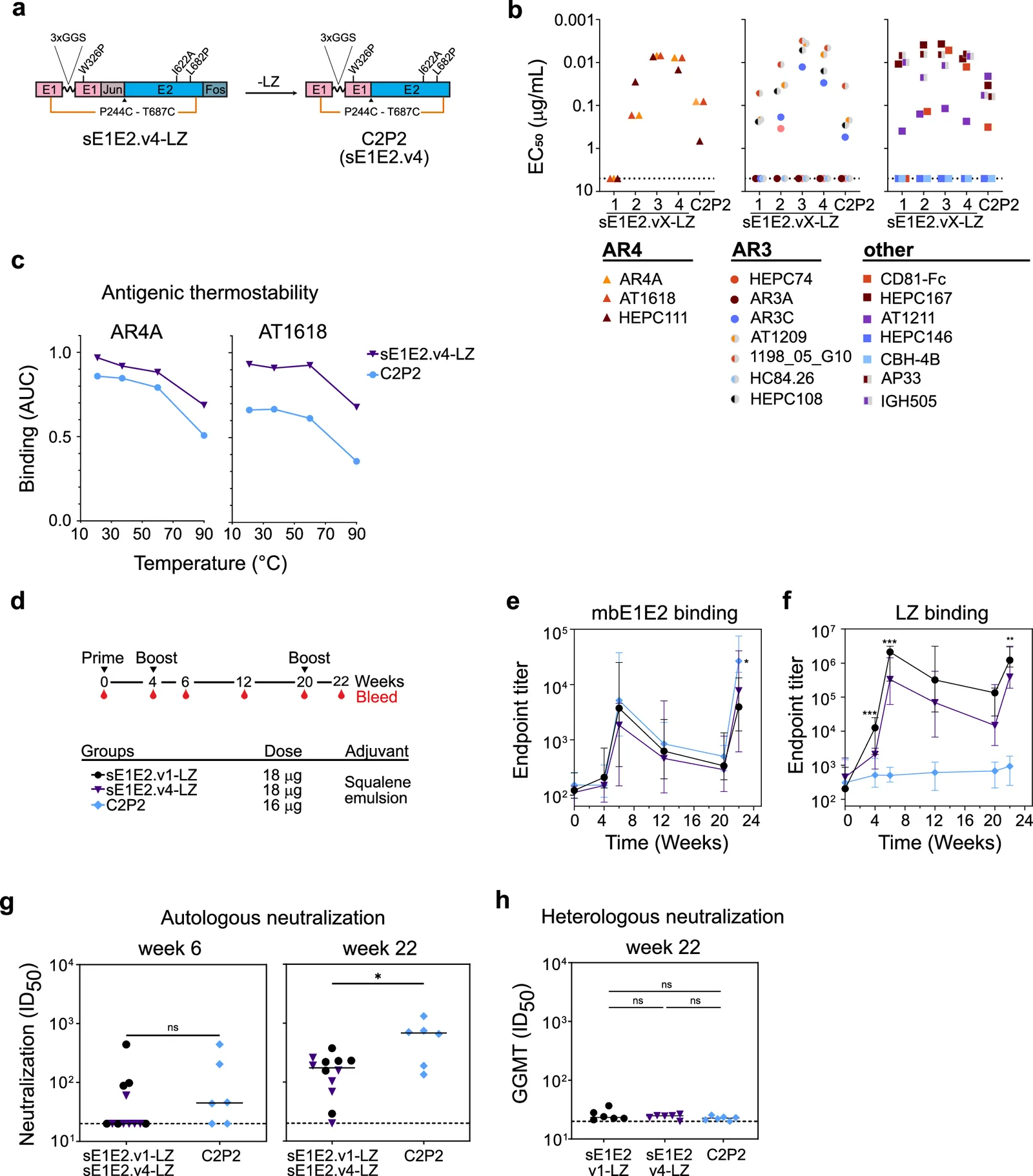
Design and immunogenicity of sE1E2 devoid of heterodimerization domains. **a** Schematic representation of C2P2. **b** ELISA results of AMS0232 C2P2 tested against a panel of 17 mAbs and CD81-Fc. EC_50_ results are plotted on the *y* axis. The results for sE1E2.v1-v4-LZ (from Fig. 1b) are included for comparison. **c** Antigenic thermostability. ELISA results showing residual binding of conformational antibodies AR4A and AT1618 to sE1E2.v4-LZ and C2P2 after being incubated at different temperatures for one h. All experiments were performed in duplicate and binding is normalized to AP33 binding at the respective temperature. **d** Study schedule in rabbits. Black triangles indicate the time points of immunization and drops indicate bleeds. Three groups of six rabbits were used. **e** ELISA endpoint titers for membrane-bound (mb) E1E2-specific IgG in rabbits. Titers between groups were compared using the Kruskal-Wallis test and correcting with Dunn’s test (p = 0.015) **f** ELISA endpoint titers for LZ-specific IgG in rabbits measured against a mix of hJun and hFos peptides. Titers between groups were compared using the Kruskal-Wallis test and correcting with Dunn’s test (*p* = 0.0009, *p* = 0.0007, *p* = 0.0011) (**g**) Neutralization titers in rabbits using the sequence-matched AMS0232 HCVpp. Titers between groups were compared using two-sided Mann-Whitney U test (*p* = 0.0415) (**h**) Breadth of the neutralizing response tested against 11 heterologous HCVpp. Global geometric mean titers (GGMT) were calculated for each rabbit serum using the individual ID_50_ values in Supplemental Figure S14. In **e**, **f** the dots represent median titers and the error bars indicate 95% confidence interval. **e**–**h**) (*p < 0.05; **p < 0.01, ***p < 0.001; ns non significant); *n* = 6 rabbit sera per group. Source data are provided as a Source Data file.

We hypothesized that the engineered 244C-687C disulfide bond in the sE1E2.v4-LZ design might facilitate heterodimerization of the E1 and E2 ectodomains without dimerization domains. To test this, we developed sE1E2.v4 by removing the LZ from sE1E2.v4-LZ (Fig. 4a). The sE1E2.v4 construct produced efficiently with a relatively high proportion of heterodimers, was efficiently cleaved (Fig. S14a) and non-reducing SDS-PAGE confirmed that E1 and E2 were covalently linked (Fig. S14b, c).

Despite the absence of a heterologous dimerization domain, sE1E2.v4 retained strong binding to AR4A, AT1618 and HEPC111, with binding levels superior to that of sE1E2.v1-LZ and comparable to sE1E2.v2-LZ (Fig. 4b). The overall antigenicity and thermal stability remained high, with AR4A binding largely retained even after incubating sE1E2.v4 for one hour at 90 °C, but lower compared to sE1E2.v4-LZ, indicating that the dimerization domains provide additional stability and/or act as folding chaperones (Fig. 4c). Additionally, the glycosylation profile of C2P2 was comparable that of sE1E2.v4-LZ (Fig. S14d). In conclusion, we successfully generated a soluble, native-like E1E2 heterodimer, that lacks dimerization domains, which we name “C2P2” for the stabilizing double cysteine (C) and double proline (P) substitutions in its design.

### AMS0232 C2P2 vaccination enhances E1E2-specific immune responses in rabbits

As a pilot study, we compared the immunogenicity of AMS0232 C2P2 to our stabilized AMS0232 sE1E2.v4-LZ and AMS0232 sE1E2.v1-LZ, which serves as a benchmark^24^, in rabbits. Six rabbits were immunized at weeks 0, 4 and 20 with 18 μg of sE1E2.v4-LZ and sE1E2.v4-LZ or 16 μg of C2P2 (i.e., equimolar amounts of E2), adjuvanted in squalene emulsion (Fig. 4d). Antibody responses were measured at weeks 0, 4, 6, 12, 20 and 22. To assess only on-target E1E2 responses, i.e., excluding Ab responses to the LZ domain, we tested Ab responses in ELISA using membrane-bound E1E2 (mbE1E2) from the AMS0232 strain. After the prime, binding titers to mbE1E2 were comparable across the three groups (Fig. 4e). After the first boost, rabbits immunized with C2P2 induced higher endpoint titers (median: 5,164) compared to those elicited by immunization with sE1E2.v1-LZ or sE1E2.v4-LZ (median: 3,743 and 1,839, respectively), although these differences did not reach statistical significance. After the second boost, binding titers mounted by C2P2 (median: 26,581) were higher than those induced by sE1E2.v1-LZ and sE1E2.v4-LZ (3,932; *p* = 0.015 and 7,733, *p* = 0.052 (Kruskal-Wallis), respectively; Fig. 4e).

Next, we measured anti-LZ responses in the sera of the rabbits. As expected, C2P2 did not mount a meaningful LZ-specific response (median: 515; Fig. 4f). In contrast, after the prime, sE1E2.v1-LZ and sE1E2.v4-LZ induced a strong LZ-specific Ab response (median titers of 10,339 and 2096, respectively), which increased significantly after the first boost (median titers of 2,114,810 and 330,297), and did not increase further after the second boost (1,219,196 and 388,791). We note that the LZ responses exceeded those against E1E2.

To assess how stabilization and/or removal of the LZ-domain affected neutralization response, we tested the serum from the rabbits against HCVpp from the autologous virus AMS0232 and a panel of heterologous HCVpps. We did not detect a significant difference between sE1E2.v1-LZ and sE1E2.v4-LZ immunized rabbits (Fig. S14d, e). Furthermore, we only detected sporadic heterologous neutralizing responses (Fig. S14d, e). However, when we compared the neutralization activity after the first boost of the two LZ-based group versus C2P2 group, we found that only 4/12 rabbit sera neutralized AMS0232 (ID_50_ titer > 20), while 4/6 rabbits from the C2P2 group neutralized AMS0232 although this difference was not statistically significant (median ID_50_ of 20 for LZ-based E1E2 versus median ID_50_ of 45 for C2P2; *p* = 0.28; Fig. 4g). After the second boost, NAb titers increased for the three groups, and we observed significantly higher autologous neutralization by rabbits immunized with AMS0232 C2P2 when compared to the pooled sE1E2.v1-LZ and sE1E2.v4-LZ group (4-fold; median ID_50_ of 680 (n = 6) versus median ID_50_ of 174 (n = 12); *p* = 0.04; Fig. 4g). However, we detected only minimal cross-neutralizing serum response after the last boost (Fig. 4h). Together, these immunogenicity results suggest that the removal of the LZ domain from AMS0232 sE1E2 might help to induce a stronger autologous neutralizing response but that this is not sufficient to induce strong cross-neutralizing responses.

## Discussion

In this study, we used an iterative structure-guided strategy to generate a panel of HCV glycoprotein antigens, including a scaffold-free native-like E1E2 design (C2P2), with more favorable antigenicity, thermostability and immunogenicity compared to previous state-of-the-art recombinant E1E2 designs^22,49^. Structural analysis of one of our constructs in complex with the AT1211 bNAb revealed an atypical epitope on E2. These native-like E1E2 mimics are a promising starting point for generation of an efficient HCV vaccine and as tools to unravel antibody responses to HCV.

The first step of our design process was replacing the hydrophobic pFP with a hydrophilic linker. As others recently reported, we also found that removal of the pFP improved antigenicity for recombinant E1E2^34^. The pFP is (partially) embedded in the membrane as confirmed by recent structural work^4^ but its function remains unclear. It is structurally conserved across the *Flaviviridae* family^9,21^ and removing it obliterates infection in target cells by HCVpp (Fig. S1f) and HCV grown in cell culture^57^, which suggests that it plays a critical functional role by using a novel membrane fusion mechanism and/or by keeping E1 closely associated with the membrane and thereby preserving E1E2 in its presumed prefusion state^9^.

The second iterative design step helped identify W326P and L682P. These are located upstream of or within putative heptad repeat regions of E1 (residues 330–347) and E2 (residues 675–699)^33,36^. Heptad repeat regions are typically a hallmark of class I fusion glycoproteins, such as those from HIV-1 and influenza virus^16^ and are key in facilitating the transition from prefusion to postfusion state of these proteins^58^. Introducing prolines in these regions prevents the formation of the six-helix bundle of the postfusion conformation^18,59–62^ and facilitates the generation of stable vaccine antigens, as exemplified by their successful application to the COVID-19 vaccines^58,62–64^. We hypothesize that 326P and 682 P impede conformational changes of E1E2 during cellular expression and/or prevent conformational sampling, and we surmise that these mutations might stabilize a putative prefusion state of E1E2. This hypothesis is further supported by the fact that 682P inhibits viral entry in the context of HCVpp^36^ and the stronger antigenic correlation between HCVpp and sE1E2.v3-LZ and sE1E2.v4-LZ compared to sE1E2.v2-LZ, which lacks 326 P and 682 P. Thus, disruption of the heptad repeat stabilizes recombinant E1E2 in a conformation that mimics the infectious E1E2 heterodimer on the virus. Further structural information, particularly of E1E2 in its postfusion state and/or on infectious virions, is needed to fully elucidate the role of these heptad repeats in HCV membrane fusion.

The pFP and proline substitutions are generalizable across different HCV genotypes and strains. Importantly, these modifications are located in the E1 and E2 stem regions which are structurally conserved across the *Hepaci-*, *Pegi- and Pestivirus* genera^9,21^. Thus, the modifications described here for HCV E1E2 could serve as a design template for generating soluble versions of E1E2 from other *Hepacivirus* species and possibly even from *Pegi-* and *Pestiviruses*, which might inform on the mechanism of this putative “class IV” of viral membrane fusion proteins.

Inspired by the recombinant designs of other viral glycoprotein antigens, we introduced the 244C-687C disulfide bond to covalently link the E1 and E2 protomers. This increased the antigenic correlation with virion-associated E1E2 by decreasing binding to several non-NAbs suggesting that the added disulfide bond helps maintain proper folding and reduces the exposure of unwanted epitopes^65,66^. Furthermore, glycosylation of the stabilized sE1E2 versions increased in comparison to sE1E2.v1-LZ. For HCV, glycans have an important role in the folding of E1E2 as revealed by both structural studies on E1E2 and by studies on virion-associated E1E2^14,67^. Although sE1E2.v4-LZ binds somewhat weaker to some bnAbs compared to sE1E2.v3-LZ, the stronger antigenic correlation with HCVpp and its weaker binding to non-NAbs indicate that sE1E2.v4-LZ is a more native-like representation of the E1E2 heterodimer. Therefore, sE1E2.v4-LZ might also be useful as a probe to isolate novel conformational bNAbs. We have already used sE1E2.v4-LZ as a tool to determine the binding stoichiometry of HCV bispecific antibodies^68^. Finally, the increased thermostability also offers significant advantages for vaccine distribution, particularly in regions where maintaining a cold chain is challenging.

The 244C-687C disulfide bond also enabled us to generate C2P2, which represents a recombinant native-like E1E2 design that lacks a stabilizing scaffold. Our proof-of-concept rabbit immunization study showed that removing the LZ scaffold seemed to redirect the antibody response towards E1E2 resulting in higher autologous (N)Ab titers despite the less favorable antigenicity compared to sE1E2.v4-LZ^55,56,69,70^. Antigen stabilization, the improved antigenicity and removal of the LZ were not sufficient to significantly improve serum cross-neutralization compared to sE1E2.v1-LZ. Similarly, stabilization and masking immunodominant epitopes on HIV-1 glycoprotein trimers redirected responses towards other epitopes but also did not enhance neutralizing breadth^71^. However, the C2P2 design presented here, can be used as a starting template to generate fusion proteins to enhance immunogenicity: either by fusing its free C-terminal end to nanoparticles for multivalent presentation^24,34,49,72–88^, or by fusing C2P2 to transmembrane domains for cell surface expression in the context of an mRNA vaccine, which may help to induce both strong B and T cell responses^89^. We did not measure how well our constructs induce T cell responses. T cells are also known to be important for protection against chronic HCV infection and should be considered in HCV vaccine development^90^. Furthermore, the immunogens were tested in rabbits, which harbor antibodies that are structurally different from human antibodies. Immunization in more relevant animal models, such as non-human primates or mice with humanized immune system, is necessary to better predict how immunogenicity of these antigens translate into humans.

We used AMS0232 sE1E2.v4-LZ to elucidate the structure of AT1211 NAb and describe a novel epitope on E2. AT1211 binds to domain C on E2 on the opposite side of the glycan shield, and is located in-between AR3 and AR4. It maximizes binding by interacting through both the HC CDR loops and through a FR3 that has undergone somatic hypermutation to improve binding. This leads to an angled approach of the antibody to the antigen, which might have evolved to accommodate the proximity of the membrane on the surface of the virus, while it also allows for binding to an otherwise hidden epitope on the glycoprotein. The intrinsic flexibility of the newly designed antigen limited how much of the complex we could solve by cryo-EM. Further work with different complexes and stabilizing antibodies will be needed to resolve the full-length construct.

For many viral glycoproteins, glycan modification has been applied to redirect the immune response and improve antigenicity^91–93^. For HIV-1, studies have shown that removing specific *N*-linked glycans can stimulate germline inferred bNAbs both in vitro and in vivo^94–97^. High-resolution structures show that some glycans on the E2 front layer are located near bNAb epitopes, or even make direct contact with them^14,40,98,99^. These glycans may hinder the initial engagement by the lower-affinity naïve germline precursor versions of these bNAbs. However, we previously found that non-native HCV glycoproteins based on the UKNP4.1.1 sequence bind some inferred germline AR3-targeting bNAbs without glycan engineering^50^. Here, we applied the stabilized designs to the UKNP4.1.1 E1E2 sequence and found that UKNP4.1.1 sE1E2.v3-LZ and sE1E2.v4-LZ engages more inferred germline bNAbs, and that binding is stronger compared to the previous non-native design. This demonstrates that native-like folding, of which AR4A bNAb binding is a proxy, also benefits the presentation of conformational epitopes of inferred germline bNAbs to AR3. The next logical steps would be to investigate whether removing and/or repositioning glycans on these native-like UKNP4.1.1-based designs enhances or broadens binding of germline precursor bNAbs and determine the effect of these stabilizing mutations on the capacity of other E1E2 sequences to engage inferred germline bNAbs.

Based on previous immunogenicity studies from us and others^34,49^, we hypothesize that a single stabilized antigen, either as a soluble protein or on a nanoparticle, will not suffice to elicit sufficient neutralizing breadth consistently. It will probably require a cocktail vaccine and/or epitope-tailored antigens administered sequentially to consistently elicit bNAbs. Our designs are compatible with strains of various genotypes and with different neutralization-sensitivities. This will enable the generation of novel native-like E1E2 antigens and vaccine strategies to develop a broad and potent B cell vaccine^49^.

Additional structure-based engineering is probably possible to further enhance expression, stability and antigenicity of our designed antigens, particularly C2P2. Lastly, native E1E2 may be arranged as a homodimer of E1E2 heterodimers^4^, and we believe the work presented here based on E1E2 heterodimers will also be useful in designing recombinant immunogen designs that aim to replicate the homodimeric arrangement of E1E2.

Overall, the recombinant HCV glycoprotein designs described here enable the creation of a broad arsenal of immunogenic HCV E1E2 antigens, which can be employed in a wide variety of applications and next-generation vaccine strategies aimed at inducing bNAbs against E1E2 epitopes.

## Methods

### Ethics statement

The rabbit animal study was performed by Labcorp (Denver, USA). Study number: 0201-23. All procedures in this study design are in compliance with the U.S. Department of Agriculture’s (USDA) Animal Welfare Act (9 CFR Parts 1, 2, and 3); the Guide for the Care and Use of Laboratory Animals (Institute of Laboratory Animal Resources, National Academy Press, Washington, D.C., 2011); and the National Institutes of Health, Office of Laboratory Animal Welfare. Whenever possible, procedures in this study are designed to avoid or minimize discomfort, distress, and pain to animals.

### Constructs

Antibody sequences used in this work were codon-optimized for humans and cloned into IgG1 expression vectors as described before^50,100,101^ (Genscript Biotech). HCV E1E2 sequences are numbered according to the standard H77 polyprotein numbering (GenBank AF009606)^102^. Constructs were based on the HCV glycoprotein sequences from the following strains (GenBank ID; mutation(s)): AMS0232 (OL855837.1; I442F), H77C (D: NP_671491.1; AAB67037; R564C, V566A, G650E), AMS3a (KR094964.1), UKNP4.1.1 (ALV85530.1). sE1E2.v1-LZ was an adapted version of the one described by Guest and his colleagues^22^. The synthetic coiled-coil SYNZIP dimerization domain was described elsewhere^54^. We previously described the UKNP4.1.1-E2E1-I53-50A construct^49^. All constructs contain a Strep-tag II to facilitate purification. All constructs were cloned into the pPPI4 expression vector and were preceded by an optimized tissue plasminogen (TPA) signal sequence to induce protein expression. All amino acid sequences are available in Supplementary Data 1. All mutations were incorporated into plasmids encoding E1E2 by means of mutagenesis. Primers were designed using the QuickChange Primer Design tool. Mutagenesis was performed using the QuikChange II (XL) Site-Directed Mutagenesis Kit (Cat# 200522-5), and the mutagenesis and transformation processes were based on the manufacturer’s protocol.

### Protein production in HEK 293T cells

Initial screenings of the mutant libraries were performed in HEK 293T cells. Cells were maintained in DMEM (Gibco)/10% fetal bovine serum (FCS)/0.1% Penicillin-Streptomycin (PS). One day prior to transfection, cells were seeded in 6-well plates at 0.3 × 10^6^ cells/well. Cells were co-transfected by addition of a mix of PEImax (1 μg/μl) with expression plasmids (5 μg DNA/well) in a ratio of protein-to-furin of 1:1, in OptiMEM. Two days laters, supernatants were harvested and mixed in 4 volumes of TBS. After centrifuging, the supernatant was used to coat Strep-TactinXT ELISA plates.

### Soluble protein production and purification in HEK 293F cells

HEK 293F (Invitrogen, cat no. R79009) cells were maintained in Freestyle medium (Life Technologies). Cells were co-transfected at a density of 0.8–1.2 million cells/mL by addition of a mix of PEImax (1 μg/μl) with expression plasmids (312.5 μg DNA/L of 293 F cells) in a 1:1 ratio (E1E2r:furin) in OptiMEM. Supernatants were harvested six days post transfection, centrifuged for 30 min at 4000 × *g* and filtered using 0.22 μm Steritop filters (Merck Millipore). The glycoproteins were purified using Strep-TactinXT columns (IBA Life Sciences) by gravity flow ( ~ 0.5–1.0 ml supernatant/min). The eluted proteins were concentrated and buffer exchanged intoTris-buffered saline (TBS, pH 7.5) using Vivaspin 6, 50,000 MWCO PES (Sartorius). Finally, purified proteins were fractionated using size-exclusion chromatography (SEC) on a Superdex 200 Increase 10/300 GL (GE Healthcare) and the fractions corresponding to E1E2 heterodimer were pooled and concentrated. The concentration was determined by Nanodrop (Thermo Fisher Scientific) using theoretical molecular weight and extinction coefficient. Aliquots of the proteins were kept at -80 °C until further use.

Similar to proteins, antibodies were expressed in HEK 293F cells. Cells were co-transfected with two plasmids expressing IgG1 heavy chain (HC) and light chain (LC) in a 1:1 ratio using 1 mg/mL PEI MAX (Polysciences) (1:3 plasmid:PEI Max). Supernatants containing the antibodies were harvested five days post-transfection, centrifuged for 30 min at 4000 × *g* and filtered using 0.22 μm Steritop filters (Merck Millipore). The filtered supernatant was run over a 1 mL protein A/G column (Pierce) followed by two column volumes of PBS wash. The antibodies were eluted with 18 mL 0.1 M glycine pH 2.5 and directly captured in 2 mL neutralization buffer (1 M TRIS pH 8.7). The purified antibodies were buffer exchanged to PBS using 100 kDa VivaSpin20 columns (Sartorius). The IgG concentration was determined using a NanoDrop 2000 and the antibodies were stored at 4 °C until further analyses.

### Membrane bound E1E2 production and purification

HEK 293T cells were transfected using 40 μg of E1E2 expression plasmids, 80 μl of Lipofectamine 2000 and Opti-MEM (Invitrogen by Thermo Fisher Scientific) per T150 flask. Three days after transfection, the supernatants were removed, and the cells were washed twice with PBS before detaching them with Trypsin-EDTA. Non-transfected HEK-293Tcells were taken along as a negative control. Cell suspensions were spun down and washed with PBS before counting them. Cells were resuspended in 1% Triton buffer (50 mM tris pH 8.0 + 150 mM NaCl + 1% Trition) with 1x proteinase inhibitor cocktail (Thermo Scientific), 1 ml per 1 × 10^6^ cells. After 30 min incubation at 4 °C with rotation, cell lysates were centrifuged at 4000 g at 4 °C for 30 min.

Membrane bound E1E2 (mbE1E2) purification was done with Galanthus Nivalis Lectin (GNL) (Vector laboratories, l-1240–5) columns. Briefly, cell lysates of transfected and non-transfected cells were diluted 1:3 with PBS and added to previously washed GNL columns, after which the columns were washed three times with PBS + tritonX100 0.1%. mbE1E2 was eluted from the column with 1.0 M alpha-d-manno-pyranoside in PBS pH 7.5. The mbE1E2 were concentrated using Vivaspin 100 kDa filters (Sartorius).

### SDS PAGE

SDS-PAGE analyses were performed as described elsewhere^66^ with some modifications. Briefly 5 µg of sE1E2 were mixed with loading dye (25 mM Tris, 192 mM Glycine, 20 % v/v glycerol, 4 % m/v SDS, 0.1 % v/v bromophenol blue in milli-Q water) and incubated at 99 °C for 10 min prior to loading on a 4–12 % Tris-Glycine gel (Invitrogen). For reducing SDS-PAGE, dithiothreitol (DTT; 100 mM) was included in the loading dye and loaded on a Novex 10–20 % Tris-Glycine gel (Thermo Fisher Scientific). Gels were run in a buffer containing 25 mM Tris, 192 mM glycine and 0.5 % SDS for over one h at 120 V at RT. Coomassie blue staining of SDS-PAGE gels was performed using the PageBlue Protein Staining Solution (Thermo Fisher Scientific).

### Strep-TactinXT ELISA

96-well Strep-TactinXT coated microplates (IBA LifeSciences) were coated with 100 μL of either 293 T supernatant or purified protein (1.8 μg/mL in TBS) for two h at room temperature. Plates were then washed three times with TBS and coated with specific bNAb diluted in casein blocking buffer (Thermo Fisher Scientific) for 90 min at room temperature. Three-fold dilution steps were used for diluting the antibody, and no antibody was added to the last well as a control. The plate was then washed three times with TBS, after which it was coated with 100 μL of HRP-labeled goat anti-human IgG (Jackson Immunoresearch) diluted in casein blocking buffer (1:3000) for 45 min at room temperature. The plate was then washed five times with TBS  +  0.05% Tween-20 and developed using develop solution [100 mM sodium acetate, 100 mM citric acid, 1% 3,3′,5,5′-tetraethylbenzidine (Sigma-Aldrich)], 0.01% H2O2. After 3.5 min the developing reaction was stopped with 0.8 M H_2_SO_4_. Light absorbance (OD450) was measured using a microplate spectrophotometer (SPECTROstar). GraphPad Prism was used to plot ELISA curves and calculate EC_50_ values.

To perform the thermostability ELISA, samples were incubated previously for one h at either 21 °C, 37 °C, 60 °C or 90 °C. Following incubation, samples were coated on Strep-TactinXT coated microplates and the same protocol as described here was followed. Similar to this, samples underwent several freeze-thaw cycles before coating them on ELISA plates.

### Site-specific glycan analysis

Two aliquots of each sample were denatured and reduced for 1 h in 50 mM Tris/HCl, pH 8.0 containing 6 M of urea and 5 mM dithiothreitol (DTT). Next, E1E2 proteins were reduced and alkylated by adding 20 mM iodoacetamide (IAA) and incubated for 1 h in the dark, followed by a 1 h incubation with 20 mM DTT to eliminate residual IAA. The alkylated E1E2 proteins were buffer exchanged into 50 mM Tris/HCl, pH 8.0, using Vivaspin columns (3 kDa) and two of the aliquots were digested separately overnight using chymotrypsin (Mass Spectrometry Grade, Promega) or alpha lytic protease (New England Biolabs) at a ratio of 1:30 (w/w). The next day, the peptides were dried and extracted using Oasis PRiME HLB µElution plate (Waters). The peptides were dried again, re-suspended in 0.1% formic acid, and analyzed by nanoLC-ESI MS with an Ultimate 3000 HPLC (Thermo Fisher Scientific) system coupled to an Orbitrap Eclipse mass spectrometer (Thermo Fisher Scientific) using stepped higher energy collision-induced dissociation (HCD) fragmentation. Peptides were separated using an EasySpray PepMap RSLC C18 column (75 µm × 75 cm). A trapping column (PepMap 100 C18 3μm particle size, 75μm × 2 cm) was used in line with the LC prior to separation with the analytical column. The LC conditions were as follows: 280-minute linear gradient consisting of 4-32% acetonitrile in 0.1% formic acid over 260 minutes followed by 20 minutes of alternating 76% acetonitrile in 0.1% formic acid and 4% ACN in 0.1% formic acid, used to ensure all the sample had eluted from the column. The flow rate was set to 300 nL/min. The spray voltage was set to 2.5 kV and the temperature of the heated capillary was set to 40 °C. The ion transfer tube temperature was set to 275 °C. The scan range was 375 − 1500 *m/z*. Stepped HCD collision energy was set to 15, 25 and 45%, and the MS2 for each energy was combined. Precursor and fragment detection were performed using an Orbitrap at a resolution MS1 = 120,000. MS2 = 30,000. The AGC target for MS1 was set to standard and the injection time was set to auto which involves the system setting the two parameters to maximize sensitivity while maintaining cycle time. Full LC and MS methodology can be extracted from the appropriate Raw file using XCalibur FreeStyle software.

Glycopeptide fragmentation data were extracted from the raw file using Byos (Version 4.6; Protein Metrics Inc.). The glycopeptide fragmentation data were evaluated manually for each glycopeptide; the peptide was scored as true-positive when the correct b and y fragment ions were observed along with oxonium ions corresponding to the glycan identified. The MS data were searched using the Protein Metrics 305 N-glycan library with sulfated glycans added manually. The relative amounts of each glycan at each site, as well as the unoccupied proportion, were determined by comparing the extracted chromatographic areas for different glycotypes with an identical peptide sequence. All charge states for a single glycopeptide were summed. The precursor mass tolerance was set at 4 ppm and 10 ppm for fragments. A 1% false discovery rate (FDR) was applied. The relative amounts of each glycan at each site, as well as the unoccupied proportion, were determined by comparing the extracted ion chromatographic areas for different glycopeptides with an identical peptide sequence. Glycans were categorized according to the composition detected.

HexNAc^2^Hex(10 + ) was defined as M9Glc, HexNAc^2^Hex(9 − 5) was classified as M9 to M3. Any of these structures containing a fucose was categorized as FM (fucosylated mannose). HexNAc^3^Hex(5 − 6)X was classified as Hybrid with HexNAc^3^Hex^5,6^Fuc^1^X classified as Fhybrid. Complex-type glycans were classified according to the number of HexNAc subunits and the presence or absence of fucosylation. As this fragmentation method does not provide linkage information, compositional isomers are grouped, so for example, a triantennary glycan contains HexNAc5, but so does a biantennary glycans with a bisect. Core glycans refer to truncated structures smaller than M3. M9Glc-M4 were classified as oligomannose-type glycans.

### HCVpp production

HCVpp generation and neutralization assays were performed as described elsewhere^103^. One day prior to transfection for generating HCVpp, 1.5 × 10^6^ HEK 293T or HEK 293T CD81KO cells were seeded on a 10 cm^2^ dish. Cells were co-transfected with three plasmids: MLV Gag-Pol packaging construct, firefly luciferase and E1E2 in optimized ratios^103,104^ with a total amount of 6 µg of DNA and 12 µl of Lipofectamine 2000 (Invitrogen) in Opti-MEM (ThermoFisher). After an incubation overnight, Opti-MEM was replaced by DMEM (Gibco)/10% FCS/0.1% PS. Two days later, the supernatant containing the HCVpps was passed through a 0.45 µm filter and frozen at −80 °C for long-term storage or 4 °C when used within a week. Infectivity of the HCVpp was done as described previously^103^.

### Rabbit immunizations

18 rabbits (New Zealand White, female, 3 groups, 6 animals/group, 2.5–3.5 kg) were immunized under subcontract at Labcorp (Denver, USA. Study No. 0201-23) with either 18 μg AMS0232 E1E2.v1, 18 μg AMS0232 E1E2.v4 or 16 μg C2P2 (equimolar to E2). Antigens were mixed 1:1 with squalene o/w emulsion (SE) (250 μL of antigen in PBS combined with 250 μL SE) (Polymun, Klosterneuburg, Austria) and administered by two intramuscular immunizations in each quadriceps (2 × 250 μL). Rabbits were bled at weeks 0, 4, 6, 12, 20 and 22. All procedures in this study design are in compliance with the U.S. Department of Agriculture’s (USDA) Animal Welfare Act (9 CFR Parts 1, 2, and 3); the Guide for the Care and Use of Laboratory Animals (Institute of Laboratory Animal Resources, National Academy Press, Washington, D.C., 2011); and the National Institutes of Health, Office of Laboratory Animal Welfare. Whenever possible, procedures in this study are designed to avoid or minimize discomfort, distress, and pain to animals.

### Neutralization assays

Huh-7 cells, a gift from François-Loїc Cosset, a hepatocyte-derived carcinoma cell line, were seeded at 1.5 × 10^4^ cells per well in a 96-well plate 24 h prior to the experiment in 100 µl DMEM/10% FCS/0.1% PS/1% nonessential amino acids/1% HEPES buffer (Huh-7 medium). HCVpps were incubated with serially diluted mAb (or serum) concentration in duplicate at 37 °C in 5% CO2. After 1 h, the HCVpp/mAb mixture (30 μL) was added to the Huh-7 cells and incubated for 4 h. After the incubation, 220 µl of Huh-7 medium was added and incubated for 72 h. After removing the media, cells were lysed and luciferase signal was measured using the Luciferase Assay System (Promega) and a GloMax luminometer (Promega, USA).

### Statistical analyses

Statistical analyses on neutralization titers (IC_50_ values) and ELISA results (EC_50_), as well as data fitting for ELISA were performed using GraphPad Prism 8.3.0. The same software was used for all other statistical analyses and data visualization. See figure legends for details on the accompanying analyses.

### Structure prediction and visualization

Molecular graphics and analyses were performed with UCSF ChimeraX, developed by the Resource for Biocomputing, Visualization, and Informatics at the University of California, San Francisco, with support from National Institutes of Health R01-GM129325 and the Office of Cyber Infrastructure and Computational Biology, National Institute of Allergy and Infectious Diseases^105,106^. For predicting the structures of full-length E1E2 including transmembrane domains AlphaFold-2.2.0 Multimer was used^25,26,107^. Code available at: https://github.com/deepmind/AlphaFold.

### Cryo-EM sample preparation and data collection

Purified sE1E2.v4 and monoclonal mabs were mixed at 1:1 mass ratio overnight at 4 °C. The next day, samples were filtered and loaded on an S200increase size-exclusion chromatography column in TBS. The complex peaks were isolated from unbound fab fractions, concentrated to a working range of 1-3 mg/mL and 3.5 μL of the concentrated samples were loaded on a glow-discharged grid (Quantifoil 1.2/1.3, Cu 300), blotted using a Vitrobot Mark IV (ThermoFisher) at 4 °C and 100% humidity and flash-frozen in liquid ethane.

Imaging was done on a Titan Glacios II (ThermoFisher) equipped with a Falcon 4(i) direct electron detector (ThermoFisher). Data was collected at a nominal pixel size of 0.718 Å ^2^ / px and a total dose of 60 electron / Å ^2^. After screening for grid quality, automated data collection was set-up with EPU (ThermoFisher) to collect 1827 movies. Movies were processed in CryoSPARC Live^108^, aligned with Patch Motion Correction and corrected with Patch CTF, rejecting micrographs with an estimated CTF above 5 Å. Blob picker was used to select particles between 60 and 160 Å. Junk particles were removed by an initial round of 2D and selected particles were taken to 3D refinement. Rounds of ab-initio model building and 3D Non-Uniform refinement jobs ensued^109^, at which point selected unbinned particles were processed to the final stage. Global and local CTF refinement was applied to the unbinned batch of final particles. The final map was generated by local refinement using a mask created in USCF ChimeraX to hide the constant region of the antibody and focus on the variable domain and antigen density. Table S1 summarizes structural and refinement statistics.

### Protein model building and refinement

The sharpened map and the antibody sequences were provided to Modelangelo^110^ for an initial model building. The resulting model was then manually refined in Coot^111^ and then real-space refined with Phenix^112^. The model was relaxed using Rosetta and then refined iteratively through Coot and Phenix. Structural figures were made using USCF ChimeraX^105^.

### Reporting summary

Further information on research design is available in the Nature Portfolio Reporting Summary linked to this article.

## Supplementary information

Supplementary information

Description of Additional Supplementary File

Reporting summary

Transparent Peer Review file

Supplementary Data 1

## Source data

Source Dataset 1

## Data Availability

The sE1E2.v4-LZ-AT1211 model has been deposited into the RSCB PDB (https://www.rcsb.org) under accession number 9Z5U and the corresponding electron microscopy data at the EMDB database (https://www.ebi.ac.uk/emdb/) under accession number EMD-73824. Mass spectrometry RAW files have been deposited on the MassIVE server (https://massive.ucsd.edu) under accession number MSV000099823. Source data are provided with this paper.
