# Supplementary material for: Structure-based design of stable recombinant hepatitis C virus E1E2 heterodimers
Source: Nat Commun. 2026 Jul 23;17:9004. doi: 10.1038/s41467-026-75744-9 (PMC13507286; doi:10.1038/s41467-026-75744-9)
Supplement: Supplementary file 1 [file 41467_2026_75744_MOESM1_ESM.pdf]

Structure-based design of stable recombinant hepatitis C virus E1E2 heterodimers

Joan Capella-Pujol[1,2†], Fabian Mulder[1,2†], Fabien Cannac[3], Stan Peters[1,2], Maddy L. Newby[4], Meliawati Poniman[1,2], Ian Zon[1,2], Wouter Olijhoek[1,2], Tim Beaumont[1,2], Max Crispin[4], Andrew B. Ward[3], Janke Schinkel[1,2], Rogier W. Sanders[1,2], Kwinten Sliepen[1,2*]

Corresponding author: k.h.sliepen@amsterdamumc.nl

The PDF file includes:

Figs. S1 to S14
Tables S1 and S2
References

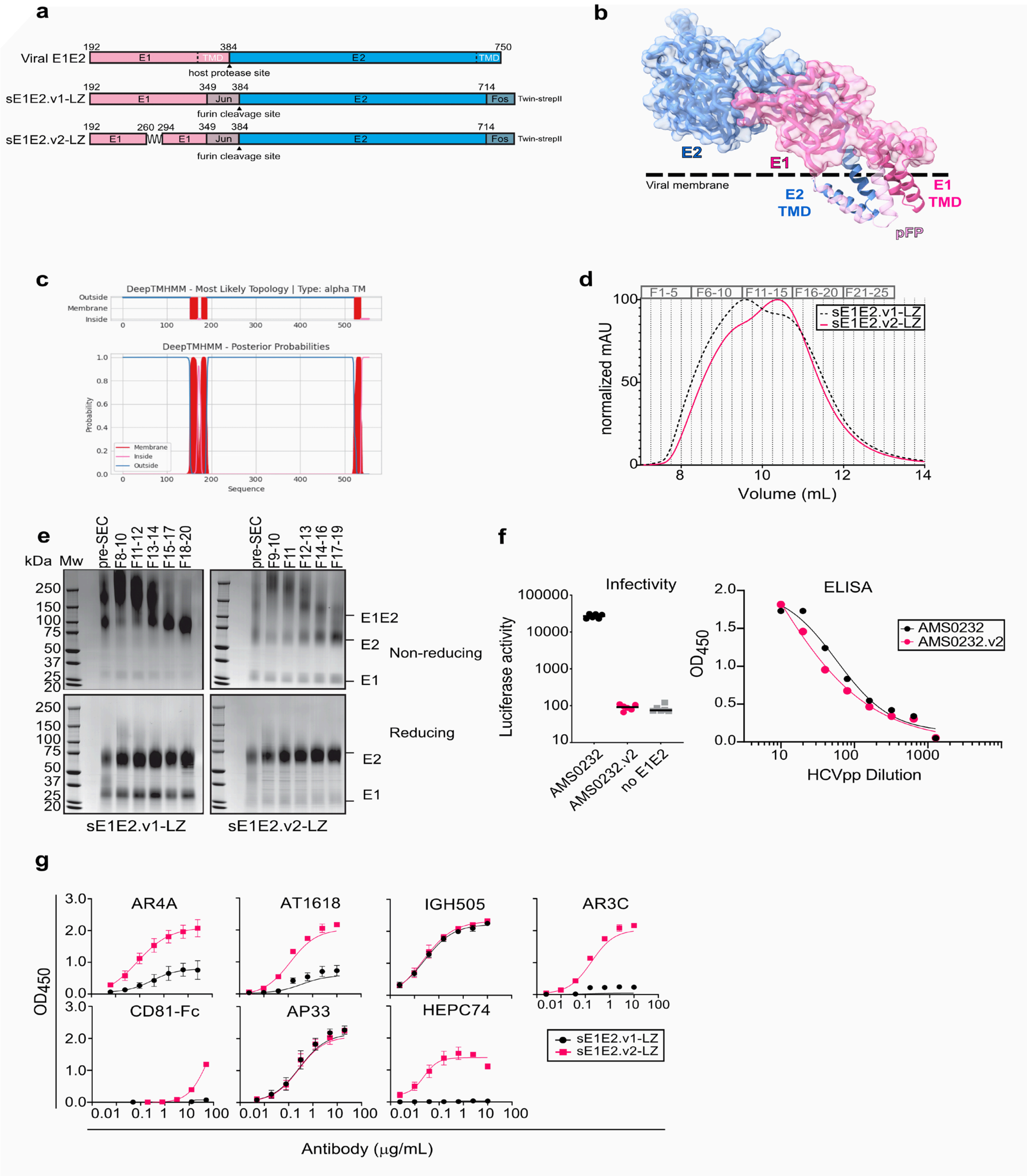

Figure S1. **Deletion of the pFP on sE1E2** (**a**) Schematic representation of sE1E2.v1-V2-LZ. (**b**) AF2 prediction overlaid with AMS0232 structure (PDB: 7T6X). Cartoons show the prediction of transmembrane domains and putative fusion peptide (pFP) to be directed towards and inside the membrane. (**c**) Transmembrane prediction of AMS0232 E1E2 full sequence by DeepTMHMM (available at https://dtu.biolib.com/DeepTMHMM). In red depicted the regions predicted to be in the membrane. E1 contains two predicted membrane regions, the transmembrane domain and one prior to that corresponding to the pFP. (**d**) SEC profiles of sE1E2.v2-LZ compared to sE1E2.v1-LZ (black dotted line). Fractions of 0.250 mL from the SEC are represented in vertical dotted lines. (**e**) Non-reducing (top) and reducing (bottom) SDS-PAGE of sE1E2.v1-LZ and sE1E2.v2-LZ of the different fractions collected from the SEC (indicated as FX, as per fractions in (**d**)) performed on the affinity purified proteins (Comassie Brilliant Blue G-250 staining). Last fractions were collected and aliquoted as purified protein. (**f**) Infectivity assay of HCVpp expressing either AMS0232 E1E2, E1E2.v2 or no E1E2 (left panel). An ELISA using the same HCVpp was used to determine the incorporation of E1E2 (bottom panel). HCVpps were diluted in TBS 1% SDS, 0.5% BSA and 50 nmol DTT and incubated 10 min at 99 °C. Samples were loaded on lectin coated plates and AP33 at single concentration (10 µg/mL) was used for detection. Replacing the pFP on membrane protein results in non-infectious HCVpp. (**g**) Representative ELISA curves using bNAbs (and CD81-Fc) targeting different epitopes against sE1E2.v1-LZ and sE1E2.v2-LZ. All experiments were performed in duplo. Mean is plotted and SEM is indicated by error bars. Source data are provided as a Source Data file.

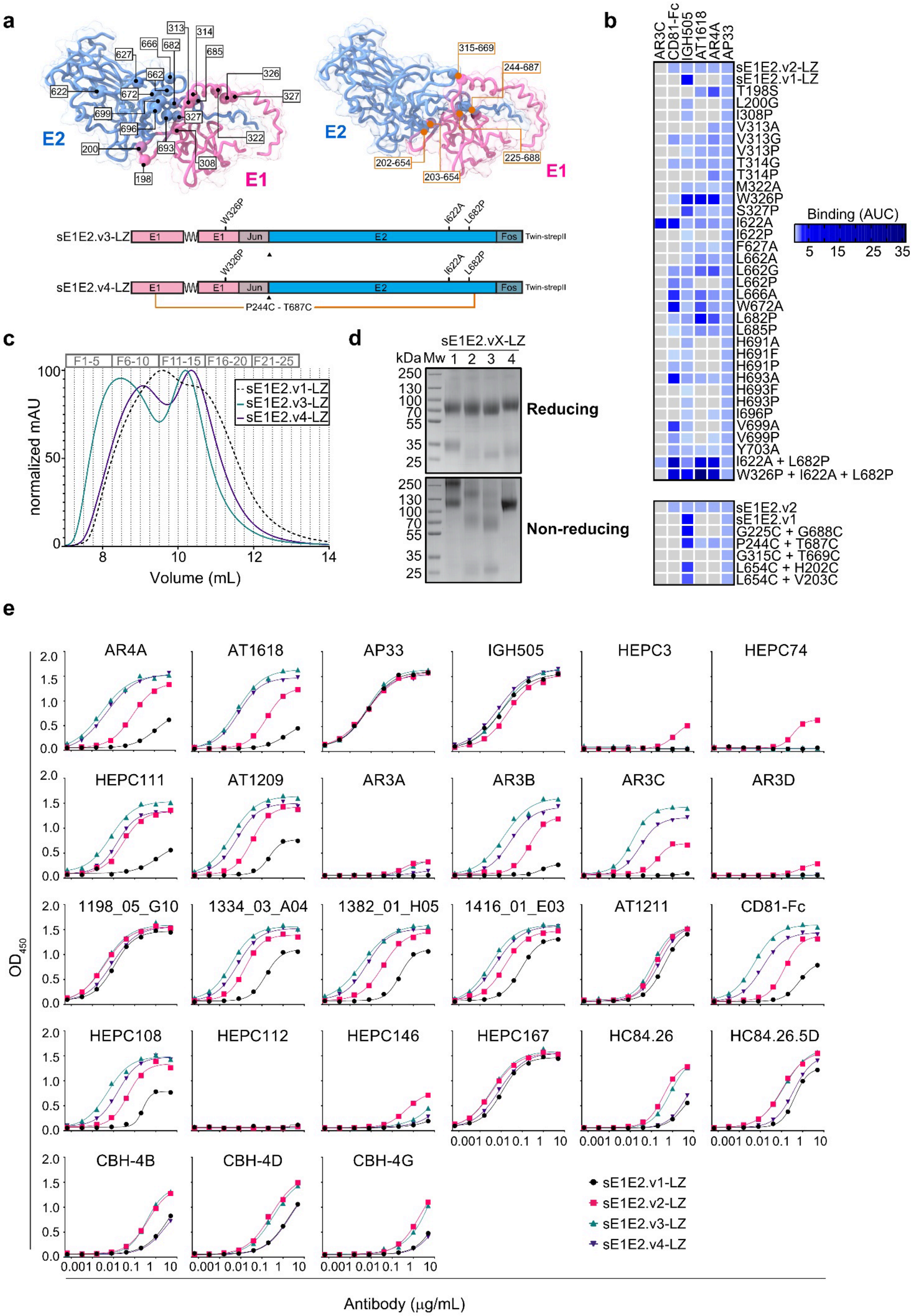
a
E2
E1
sE1E2.v3-LZ
sE1E2.v4-LZ
W326P
I622A
L682P
Jun
Fos
Twin-strepII
P244C - T687C
b
AR3C
CD81-Fc
IGH505
AT1618
AR4A
AP33
Binding (AUC)
c
F1-5
F6-10
F11-15
F16-20
F21-25
sE1E2.v1-LZ
sE1E2.v3-LZ
sE1E2.v4-LZ
normalized mAU
Volume (mL)
d
sE1E2.vX-LZ
kDa
Mw
Reducing
Non-reducing
e
OD450
Antibody (μg/mL)
sE1E2.v1-LZ
sE1E2.v2-LZ
sE1E2.v3-LZ
sE1E2.v4-LZ

Figure S2. **Design and testing of stabilizing mutations** (**a**) Schematic representation of all positions that were mutated and screened for AR4A binding improvement. Point mutations are indicated as dots over the cryo-EM structure of E1E2 (PDB: 7T6X). The left panel is the panel of mutations tested for improvement of AR4A and the right depicts the cysteines introduced in E1 and E2 to form a new disulfide bond. (**b**) Heatmap summarizing all the ELISA results from 293T supernatant screening. Binding is represented as area under the curve (AUC) and normalized to sE1E2.v2-LZ and AP33 (to normalize loading). Darker blue indicates higher binding and gray indicates loss of binding (<50% than v2) or no binding. Top panel is for the point mutations and bottom panel is for the cysteines introduced. (**c**) SEC profiles of sE1E2.v3-LZ and .v4-LZ compared to sE1E2.v1-LZ. Fractions of 0.250 mL from the SEC are represented in vertical dotted lines (**d**) Reducing and non-reducing SDS-PAGE of purified sE1E2.v1-v4-LZ (Comassie Brilliant Blue G-250 staining). (**e**) ELISA curves of all monoclonal antibodies and CD81-Fc tested against purified sE1E2.v1-LZ to sE1E2.v4-LZ. Experiments performed in duplo, mean values are depicted. Source data are provided as a Source Data file.

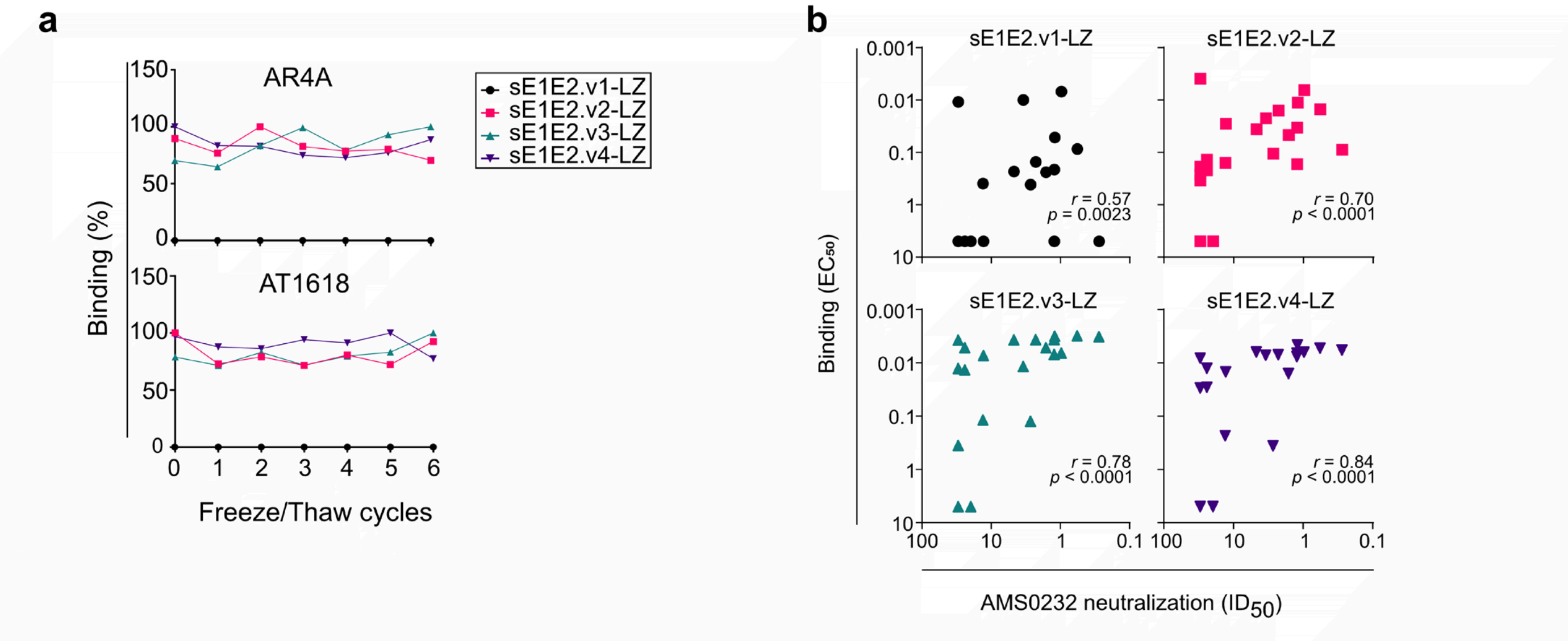

Figure S3. Characterization of sE1E2.v1-LZ-v4 (**a**) Binding to sE1E2.v1-v4-LZ after N cycles of freezing and thawing the samples. Binding (AUC) is normalized to the highest value of the set and expressed as percentage. (**b**) AMS0232 HCV pseudoparticles were tested against the same 26 mAb panel as in Fig. 1B. Each dot represents one of the 26 mAbs with its corresponding neutralization ID_{50} value on the x-axis and binding EC_{50} on the y-axis. Spearman *r* and *p* values are depicted. Values from at least two independent experiments performed in duplo or triplo. Source data are provided as a Source Data file.

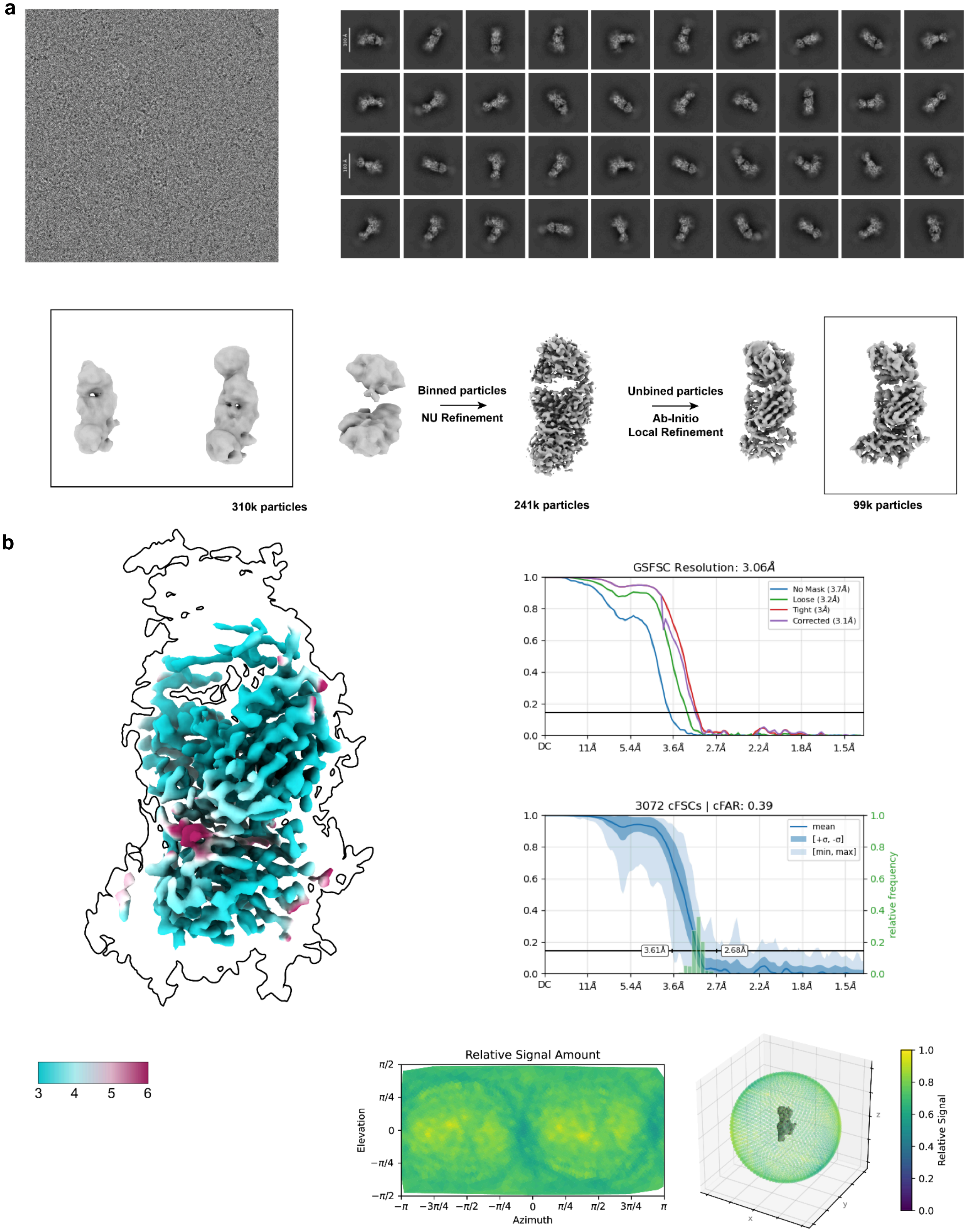

Figure S4. Cryo-EM Structural analysis of AT1211-bound E1E2. (a) Representative micrograph at around -1 µm defocus (left) and representative 2D classes of the complex (right). Ab-initio reconstruction and non-uniform refinement volumes shown below, particles were down-selected until a final set of 98 928 particles that were locally refined as shown in B. **(b)** Cryo-EM map showing the full density as a contour and the high-resolution density from local refinement as volume, colored by local resolution. Gold-Standard Fourier Shell Correlation (GS-FSC, top), conical Fourier Shell Correlation (cFSC, middle) with a line at the 0.143 gold standard criterion, and relative signal distribution across all viewing directions (bottom) shown on the right.

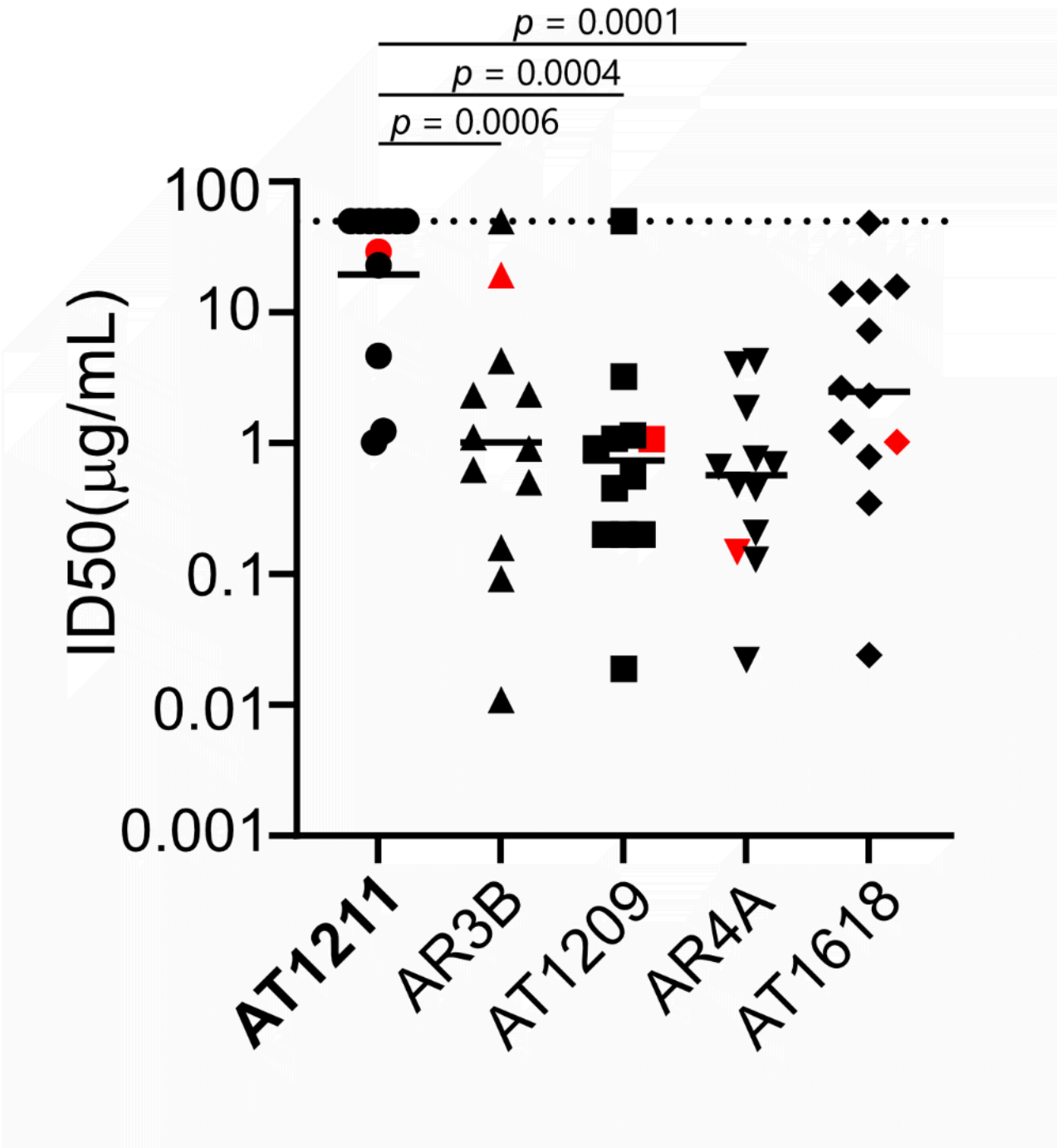

Figure S5. AT1211 neutralization data. Compiled data for the neutralization of AT1211 against a panel of HCVpp. AMS0232 data is highlighted in red. Data extracted from Chumbe et al 2024(*1*). Friedman test. Source data are provided as a Source Data file.

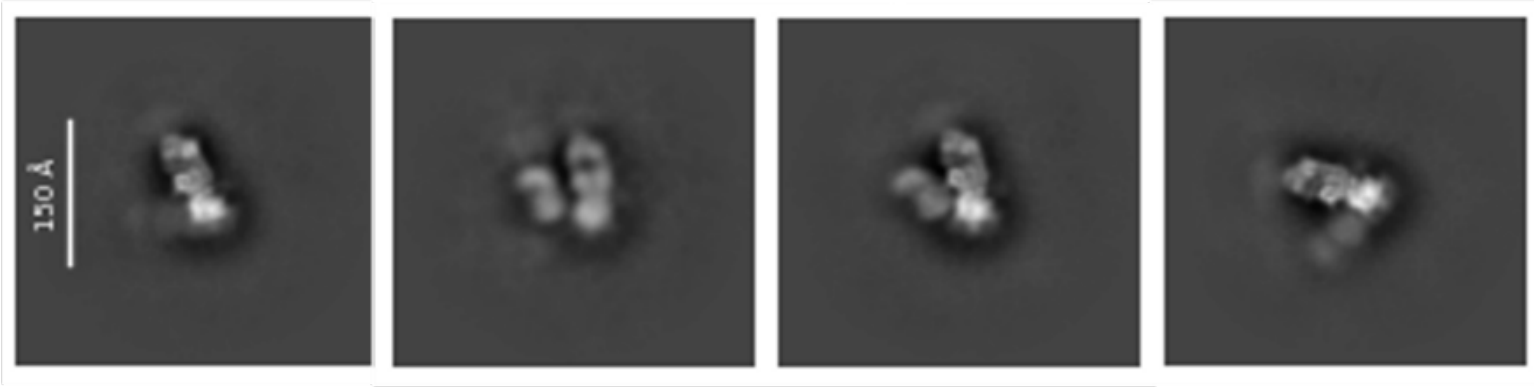

Figure S6. Cryo-EM class averages of IGH505+AT1211-bound E1E2. Selected 2D classes of a complex of E1E2 with IGH505 (binding E1) and AT1211 (binding E2), with two distinct Fab molecules visible despite a high degree of relative flexibility.

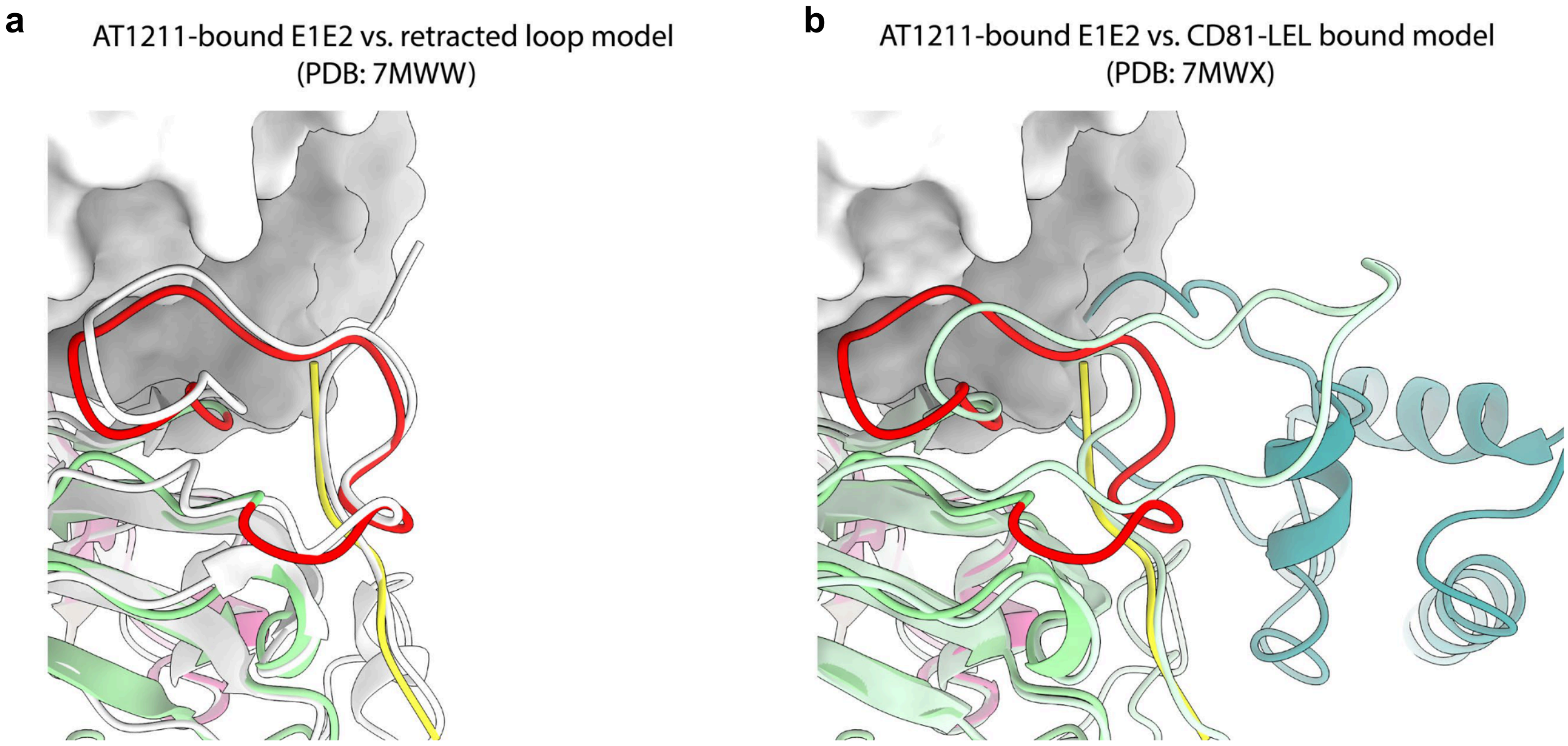

Figure S7. Comparison of unbound and CD81-bound E2 models. (**a**) Overlay of AT1211-bound E2 with previously solved model showing the CD81-binding loop in a retracted position (white, PDB: 7MWW). Color code as previously described. AT1211 is shown as surface. (**b**) Overlay of AT1211-bound E2 with previously solved model of CD81-LEL-bound E2 (PDB: 7MWX). CD81-LEL is colored teal.

```
                                                CDRH1                   CDRH2                                       CDRH3
                           10        20        30        40        50   54 57 60        70        80                95         103
IGHV1-69*18       QVQLVQSGAEVKKPGSSVKVSCKASGGTFSSYAISWVRQAPGQGLEWMGRIIPIFGTANYAQKFQGRVTITADESTSTAYMELSSLRSEDTAVYYCAR
gl-AT1211 HC      ..................................................................................................HDYVWGTPLDIWGQGTMVTVSS
AT1211 HC         ..............................G.G.T..............T...VSA.ET...RL......S...HST.S...V...K.....L..........................
```

Figure S8. **Sequence alignment of inferred germline and mature AT1211 HC**. Sequences are aligned to IGHV1-69*18 and numbered using Kabat numbering

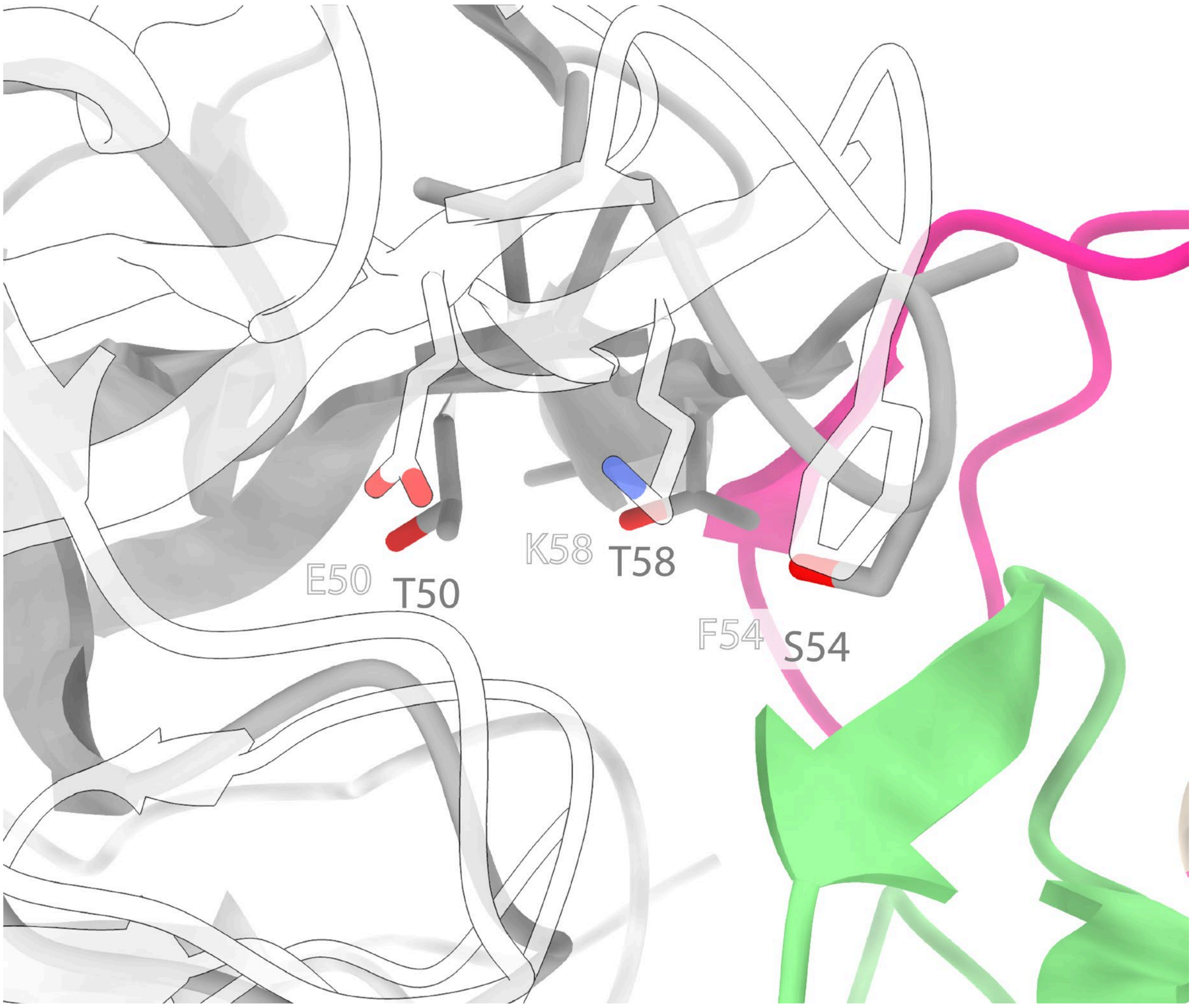

Figure S9. Comparison to CBH7-bound model. Overlay of AT1211-bound E2 with previously solved model bound to CBH7 mAb (transparent with black outline, PDB: 8THZ). Color code as previously described. Amino acids numbering is following Kabat convention.

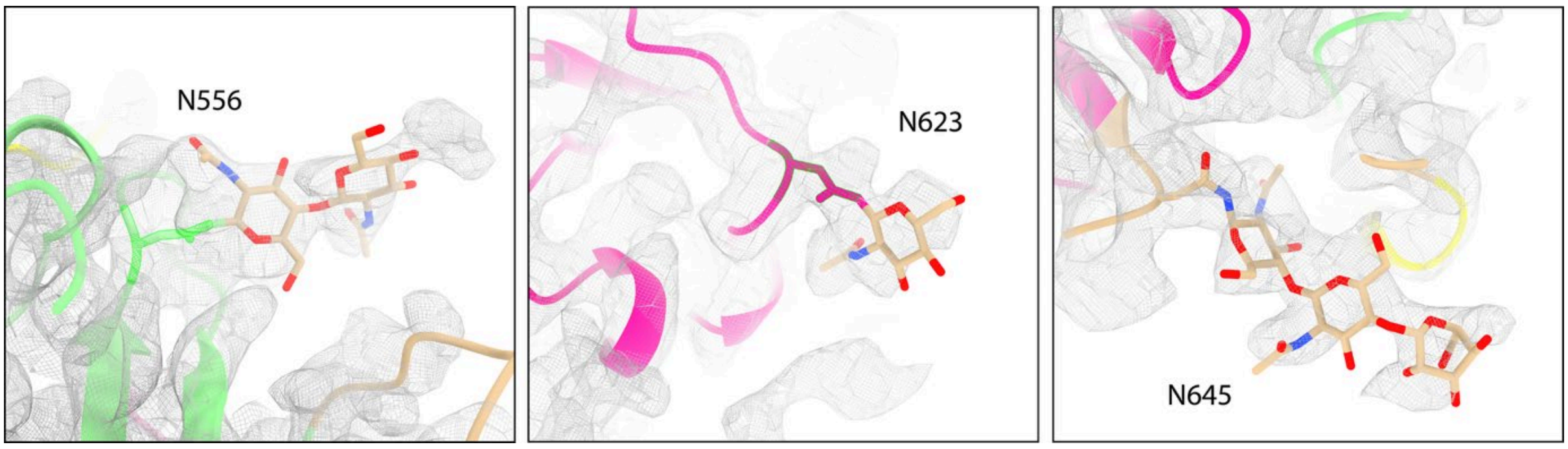

Figure S10. Glycosylations in the sE1E2.v4-LZ + AT1211 cryo-EM map. Clear density is visible for post-translational modifications at Asn 556, Asn 623 and Asn 645. For each respective position, a molecule of NAG-NAG, NAG, or NAG-NAG-BMA glycan was modelled (NAG: N-acetyl-β-d-glucosamine; BMA: β-d-mannose).

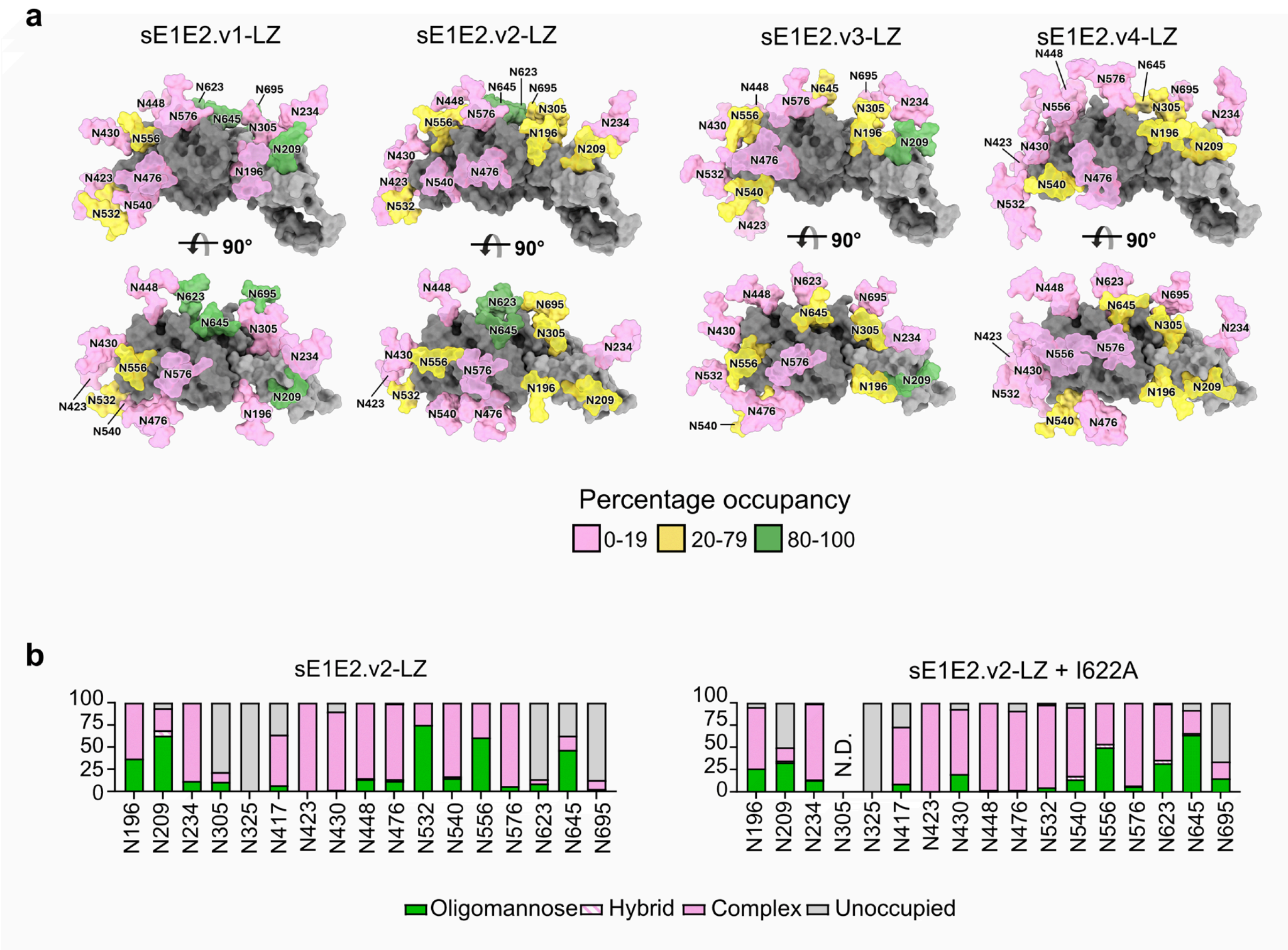

Figure S11. Site-specific glycan analysis. (**a**) Modelled glycan occupancy of the protein sE1E2.v1-v4-LZ on E1E2 (PDB: 7T6X) (**b**) Relative quantification of distinct glycan types of the proteins sE1E2.v2-LZ without and with I622A mutation was determined by LC-MS. The bar graphs show the relative percentage of the glycan processing state at a particular site. Oligomannose-type glycans are shown in green, hybrid in dashed pink, complex glycans in pink and unoccupied sites in gray. N.D. stands for not determined. Source data are provided as Source Data file.

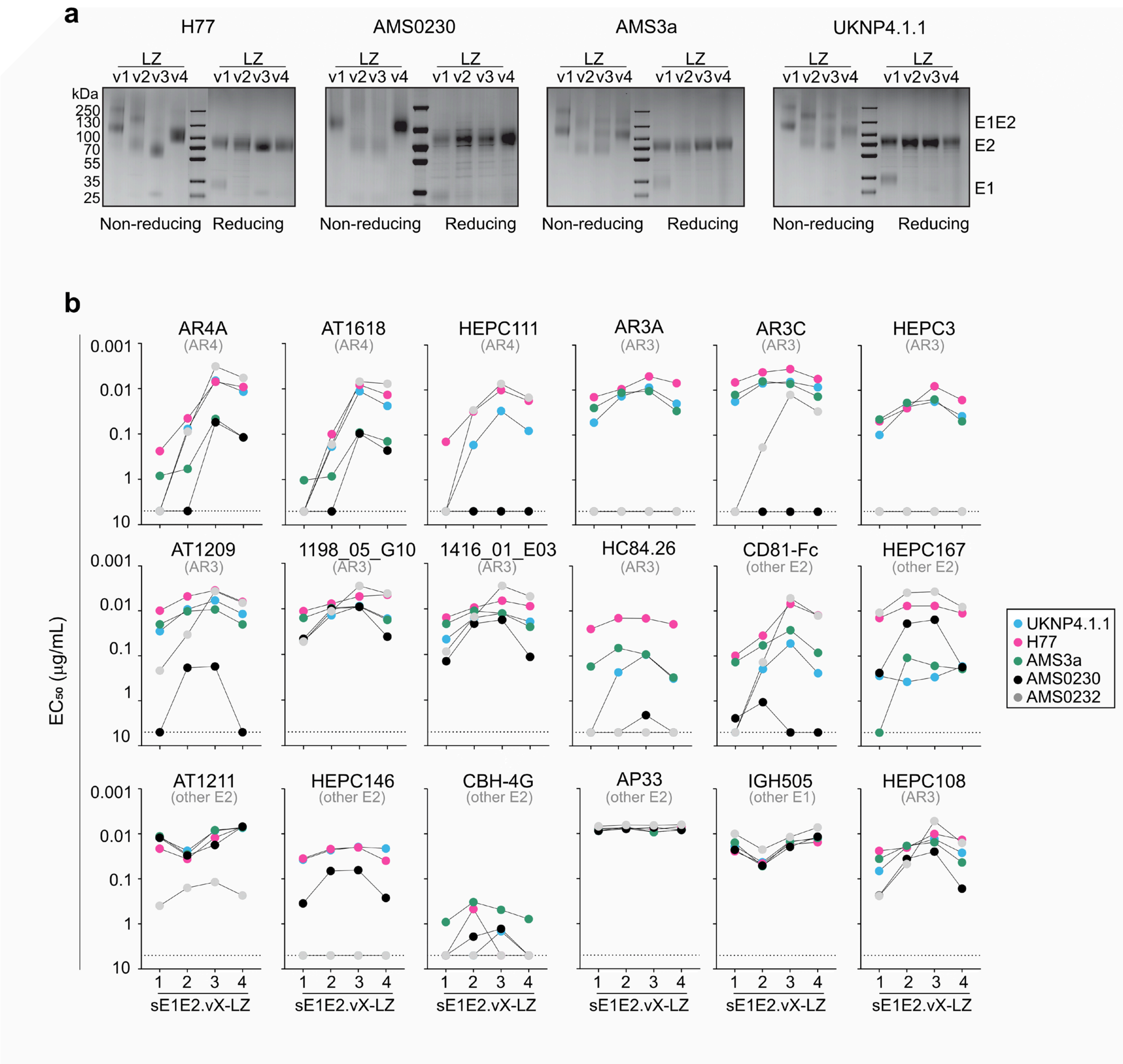

Figure S12. Stabilizing mutations are generalizable to other HCV viral strains (**a**) Versions 1 to 4 were designed with the sequences from strains H77 (genotype 1a), AMS3a (genotype 3a), UKNP4.1.1 (genotype 4a) and AMS0230 (genotype 1a). 5 µg of protein were loaded to gels in reducing and non-reducing conditions. The newly formed disulfide bond is observed in all constructs. (**b**) Pooled results from all ELISA experiments of antibodies tested against sE1E2.v1-LZ-v4 from different strains. Plotted are the EC_{50} values obtained from the analysis of two independent experiments. Dotted line represents the threshold set for these experiments, which is a maximum concentration used of antibody of 5 µg/mL. Same value (EC_{50}=5 µg/mL) was given to antibodies for which a full curve could not be obtained using this concentration. All experiments performed in duplo. Source data are provided as a Source Data file.

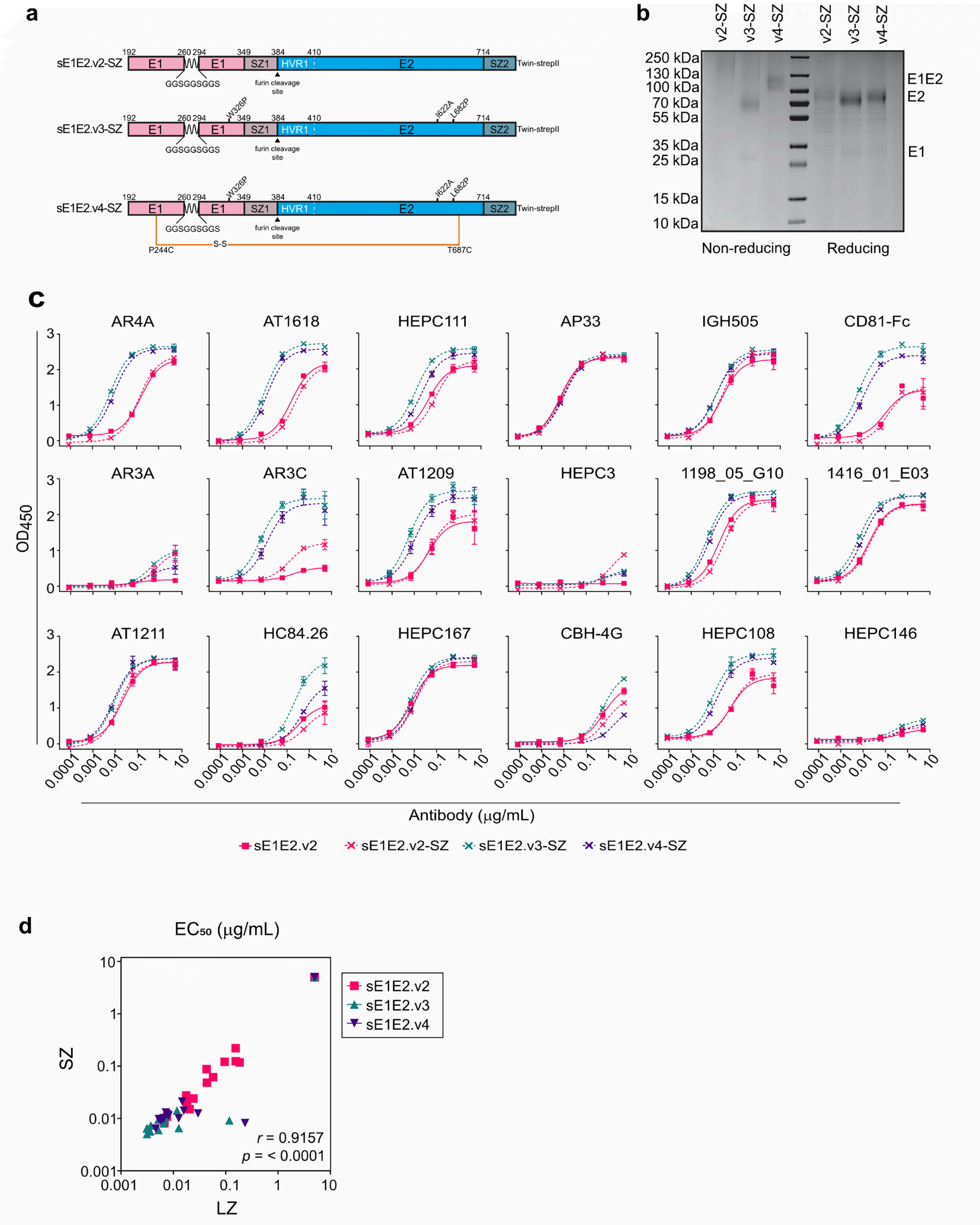

Figure S13. Heterodimerization domains Jun/Fos are interchangeable with other coiled-coil dimerization domains (**a**) Schematic design of v2-v4 with the synthetic leucine zipper SYNZIP as dimerization domains. (**b**) Reducing and non-reducing gels of sE1E2-SZ v2-v4. About 5 µg of protein were loaded on to 4-20% tris-glycine gels after boiling for ten minutes in reducing and non-reducing conditions. Gel was stained with PageBlue Protein Staining Solution. v4 shift in size is observed in non-reducing gel confirming the newly formed disulfide bond. (**c**) Full ELISA curves from a selected panel of 17 mAbs and CD81-Fc tested. All experiments performed in duplo. Data are presented as mean values +/- SEM. (**d**) The Jun/Fos dimerization domains from sE1E2.v2-LZ-v4 were swapped for the synthetic coiled-coil SYNZIP1/SYNZIP2 (sE1E2-Syn/Zip). ELISA data (EC_{50} values in µg/mL) from a selected panel of 17 mAbs and CD81-Fc are compiled and represented on the x axis for the sE1E2 versions with Jun/Fos and on the y axis the same constructs utilizing the Syn/Zip heterodimerization domains. All experiments to determine EC_{50} values were performed in duplo. Spearman rank correlation (r) was calculated to determine correlation between values. Source data are provided as a Source Data file.

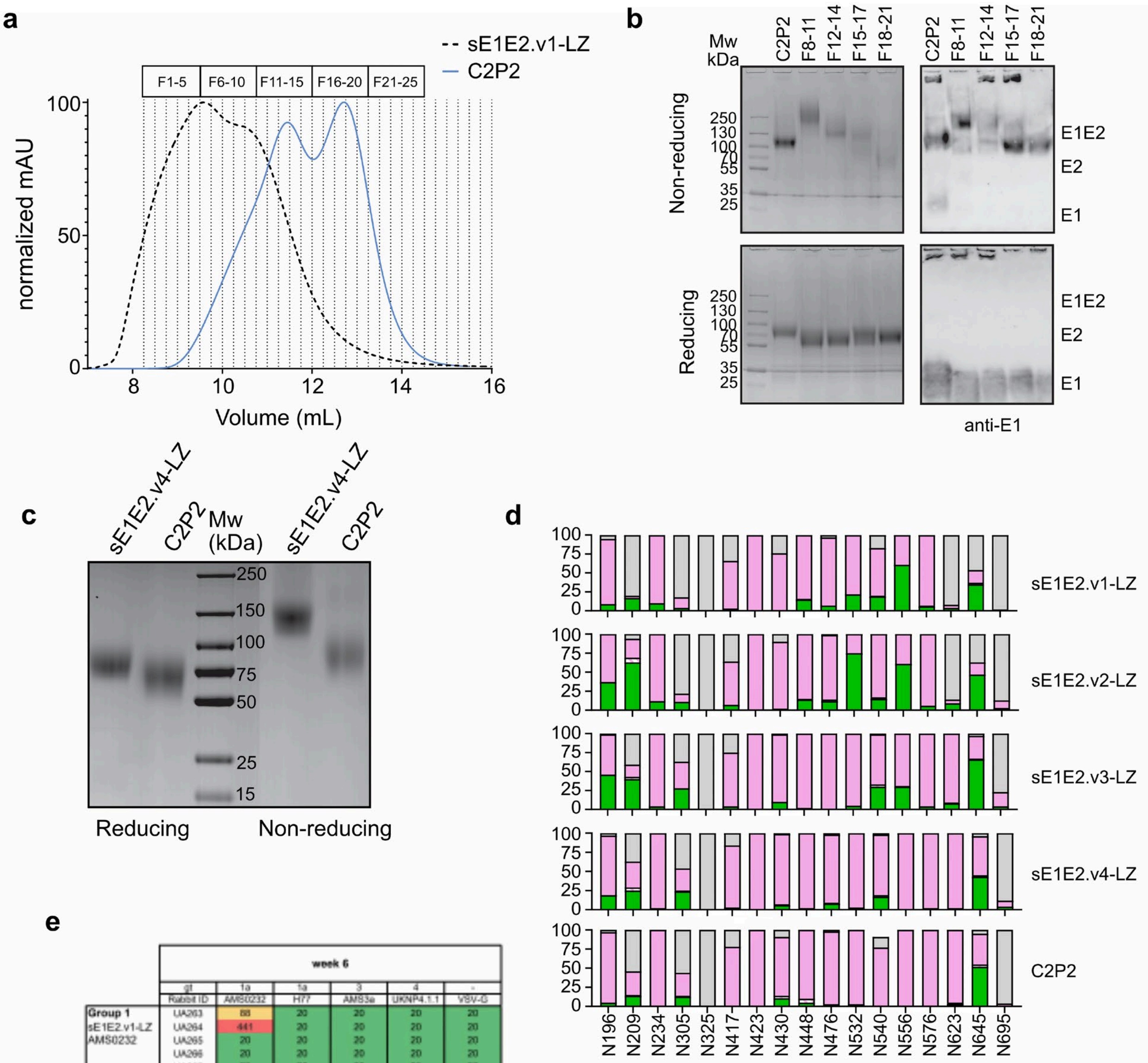

e

	week 6					
	gt	1a	1a	3	4	-
	Rabbit ID	AMS0232	H77	AMS3a	UKNP4.1.1	VSV-G
Group 1	UA263	88	20	20	20	20
sE1E2.v1-LZ	UA264	441	20	20	20	20
AMS0232	UA265	20	20	20	20	20
	UA266	20	20	20	20	20
	UA267	20	20	20	20	20
	UA268	98	20	20	20	20
Group 2	UA269	20	20	20	20	20
sE1E2.v4-LZ	UA270	20	20	20	20	20
AMS0232	UA271	60	20	20	20	20
	UA272	20	20	20	20	20
	UA273	20	20	20	20	20
	UA274	20	20	20	20	20
Group 3	UA275	46	20	20	20	20
C2P2	UA276	20	20	20	20	20
AMS0232	UA277	443	20	20	20	20
	UA278	20	20	20	20	20
	UA279	204	20	20	20	20
	UA280	44	20	20	20	20

f

	week 22													
	gt	1a	1a	3	4	2	2	2	1	5	-	1a	2	4
	Rabbit ID	AMS0232	H77	AMS3a	UKNP4.1.1	UKNP2.4.1	JFH1	gt2b	AMS0230	UKNP5.2.1	VSV-G	AMS0229	UKNP2.2.1	AMS4d.k9
Group 1	UA263	231	20	20	20	20	32	29	95	20	20	45	20	33
sE1E2.v1-LZ	UA264	220	20	20	20	20	20	20	48	20	20	30	20	20
AMS0232	UA265	29	20	20	20	20	20	20	41	20	20	32	20	22
	UA266	228	20	20	26	20	57	62	75	96	20	45	35	20
	UA267	375	20	20	20	20	20	20	20	20	20	29	20	28
	UA268	156	20	20	20	20	20	20	149	20	20	20	20	20
Group 2	UA269	103	20	20	23	20	111	20	28	20	20	22	20	20
sE1E2.v4-LZ	UA270	259	20	20	20	20	49	20	40	20	20	21	20	20
AMS0232	UA271	191	20	20	20	20	114	20	20	20	20	41	20	20
	UA272	157	20	20	20	20	313	20	27	20	20	20	20	20
	UA273	69	20	20	20	20	57	20	84	20	20	20	20	20
	UA274	20	20	20	20	20	20	20	20	20	20	20	20	20
Group 3	UA275	656	20	20	20	20	20	20	20	20	20	27	28	20
C2P2	UA276	134	20	20	20	20	20	20	20	20	20	20	20	122
AMS0232	UA277	189	20	20	20	20	20	20	20	20	20	20	20	20
	UA278	743	20	20	20	20	62	20	20	20	20	20	20	20
	UA279	704	20	20	21	20	93	20	60	20	20	20	20	20
	UA280	1316	20	20	20	20	20	20	46	20	20	44	20	20

Figure S14. Characterization and immunogenicity of a soluble native-like E1E2 free of dimerization domains (**a**) Chromatogram of purified C2P2 (blue) compared to sE1E2.v1-LZ (black). Vertical lines indicate collection fractions and in boxes the number of the fractions collected used for later characterization with protein gels. (**b**) Non-reducing (top) and reducing (bottom) SDS-PAGE gels of different fractions collected on the SEC as in (**a**) after C2P2 purification. Same gels were used for transfer of proteins into a PVDF membranes and IGH505 (anti-E1) was used for E1 detection. (**c**) SDS-PAGE gel comparing side by side AMS0232 sE1E2.v4-LZ and C2P2. 5 µg of protein were loaded and the gel was stained with Protein Staining Solution. Difference in size due to the removal of the heterodimerization domains can be observed both in reducing (left) and nonreducing (right) gels. (**d**) Relative quantification of distinct glycan types of the proteins sE1E2.v1-v4-LZ and C2P2 was determined by LC-MS. The bar graphs show the relative percentage of the glycan processing state at a particular site. Oligomannose-type glycans are shown in green, hybrid in dashed pink, complex glycans in pink and unoccupied sites in gray. (**e**) – (**f**) Midpoint neutralization (ID_{50}) titers for sera from rabbits immunized with different recombinant HCV glycoprotein antigens. Sera from week 6 (**e**), two weeks post first boost, and week 22 (**f**), two weeks post second boost, were tested against a panel of HCV pseudoparticles in standard neutralization assay. Green indicates no neutralization and red indicates highest titers and therefore strongest neutralization. Source data are provided as a Source Data file.

Table S1. **Cryo-EM data collection, refinement and validation statistics.**

	(EMDB-73824)<br>(PDB 9Z5U)
**Data collection and processing**	
Magnification	190x
Voltage (kV)	200
Electron exposure (e–/Å^2)	60
Defocus range (μm)	-0.8 to -1.8
Pixel size (Å)	0.718
Symmetry imposed	C1
Initial particle images (no.)	1 149 062
Final particle images (no.)	98 982
Map resolution (Å)	2.97
FSC threshold	0.143
**Refinement**	
Map sharpening *B* factor (Å^2)	-89.5
Model composition	
Non-hydrogen atoms	3348
Protein residues	423
Ligands	7
B factors (Å^2)	
Protein	84.79
Ligand	124.61
R.m.s. deviations	
Bond lengths (Å)	0.005
Bond angles (°)	0.934
Validation	
MolProbity score	1.78
Clashscore	6.9
Poor rotamers (%)	0
Ramachandran plot	
Favored (%)	93.98
Allowed (%)	6.02
Disallowed (%)	0

References

1. A. Chumbe, M. Grobben, J. Capella-Pujol, S. M. Koekkoek, I. Zon, S. Slamanig, S. J. Merat, T. Beaumont, K. Sliepen, J. Schinkel, M. J. van Gils, A panel of hepatitis C virus glycoproteins for the characterization of antibody responses using antibodies with diverse recognition and neutralization patterns. *Virus Res.* **341**, 199308 (2024).
