# Supplementary material for: Structure-based design of stable recombinant hepatitis C virus E1E2 heterodimers
Source: Nat Commun. 2026 Jul 23;17:9004. doi: 10.1038/s41467-026-75744-9 (PMC13507286; doi:10.1038/s41467-026-75744-9)
Supplement: Supplementary file 2 [file 41467_2026_75744_MOESM2_ESM.pdf]

Description of the Additional Supplementary Files

Supplementary Data 1: Amino acid sequences of the recombinant E1E2 constructs used in this study
