# Supplementary material for: Structure-based design of stable recombinant hepatitis C virus E1E2 heterodimers
Source: Nat Commun. 2026 Jul 23;17:9004. doi: 10.1038/s41467-026-75744-9 (PMC13507286; doi:10.1038/s41467-026-75744-9)
Supplement: Supplementary file 3 [file 41467_2026_75744_MOESM4_ESM.pdf]

nature portfolio

Corresponding author(s): Kwinten Sliepen

Last updated by author(s): Jun 30, 2026

Reporting Summary

Nature Portfolio wishes to improve the reproducibility of the work that we publish. This form provides structure for consistency and transparency in reporting. For further information on Nature Portfolio policies, see our Editorial Policies and the Editorial Policy Checklist.

Statistics

For all statistical analyses, confirm that the following items are present in the figure legend, table legend, main text, or Methods section.

n/a	Confirmed	
☐	☒	The exact sample size (n) for each experimental group/condition, given as a discrete number and unit of measurement
☐	☒	A statement on whether measurements were taken from distinct samples or whether the same sample was measured repeatedly
☐	☒	The statistical test(s) used AND whether they are one- or two-sided<br>*Only common tests should be described solely by name; describe more complex techniques in the Methods section.*
☐	☒	A description of all covariates tested
☐	☒	A description of any assumptions or corrections, such as tests of normality and adjustment for multiple comparisons
☐	☒	A full description of the statistical parameters including central tendency (e.g. means) or other basic estimates (e.g. regression coefficient) AND variation (e.g. standard deviation) or associated estimates of uncertainty (e.g. confidence intervals)
☒	☐	For null hypothesis testing, the test statistic (e.g. F, t, r) with confidence intervals, effect sizes, degrees of freedom and P value noted<br>*Give P values as exact values whenever suitable.*
☒	☐	For Bayesian analysis, information on the choice of priors and Markov chain Monte Carlo settings
☒	☐	For hierarchical and complex designs, identification of the appropriate level for tests and full reporting of outcomes
☐	☒	Estimates of effect sizes (e.g. Cohen's d, Pearson's r), indicating how they were calculated

Our web collection on statistics for biologists contains articles on many of the points above.

Software and code

Policy information about availability of computer code

Data collection	We used Microsoft Excel 2016 (16.05356.1000) for data storage. Mass spectrometry data were acquired using Xcalibur (version 4.7, ThermoFisher Scientific)
Data analysis	Graphpad Prism 8.3, Microsoft Excel 2016 (16.05356.1000), Power Point 2016 (16.05356.1000), Affinity (4231), AlphaFold-2.2.0 Multimer, UCSF ChimeraX (version 1.19), Protein Metrics (version 4.6), CryoSPARC (4.7.1), Modelangelo (1.0.12), Coot (0.9.8.92), Phenix (1.21.1-5286)

For manuscripts utilizing custom algorithms or software that are central to the research but not yet described in published literature, software must be made available to editors and reviewers. We strongly encourage code deposition in a community repository (e.g. GitHub). See the Nature Portfolio guidelines for submitting code & software for further information.

Data

Policy information about availability of data

All manuscripts must include a data availability statement. This statement should provide the following information, where applicable:

- Accession codes, unique identifiers, or web links for publicly available datasets
- A description of any restrictions on data availability
- For clinical datasets or third party data, please ensure that the statement adheres to our policy

The sE1E2.v4-LZ-AT1211 model has been deposited into the RSCB PDB (https://www.rcsb.org) under accession number 9Z5U and the corresponding electron

microscopy data at the EMDB database (https://www.ebi.ac.uk/emdb/) under accession number EMD-73824. Mass spectrometry RAW files have been deposited on the MassIVE server (https://massive.ucsd.edu) under accession number MSV000099823. Source Data are provided with this paper.

Research involving human participants, their data, or biological material

Policy information about studies with human participants or human data. See also policy information about sex, gender (identity/presentation), and sexual orientation and race, ethnicity and racism.

Reporting on sex and gender	N/A see below
Reporting on race, ethnicity, or other socially relevant groupings	N/A see below
Population characteristics	N/A see below
Recruitment	N/A see below
Ethics oversight	N/A

Note that full information on the approval of the study protocol must also be provided in the manuscript.

Field-specific reporting

Please select the one below that is the best fit for your research. If you are not sure, read the appropriate sections before making your selection.

☒ Life sciences ☐ Behavioural & social sciences ☐ Ecological, evolutionary & environmental sciences

For a reference copy of the document with all sections, see nature.com/documents/nr-reporting-summary-flat.pdf

Life sciences study design

All studies must disclose on these points even when the disclosure is negative.

Sample size	The number of animals in the study is the minimum required to provide sufficient statistical power and observe differences between treatment groups.
Data exclusions	No data was excluded
Replication	All experiments were perfromed in duplo or triplo and-or repeated at least twice. All attempts to repeat the experiments were successful.
Randomization	No randomization could be applied the output of the experiments could not be biased.
Blinding	Blinding was not relevant for our studies

Reporting for specific materials, systems and methods

We require information from authors about some types of materials, experimental systems and methods used in many studies. Here, indicate whether each material, system or method listed is relevant to your study. If you are not sure if a list item applies to your research, read the appropriate section before selecting a response.

Materials & experimental systems

n/a	Involved in the study	
☐	☒	Antibodies
☐	☒	Eukaryotic cell lines
☒	☐	Palaeontology and archaeology
☐	☒	Animals and other organisms
☒	☐	Clinical data
☒	☐	Dual use research of concern
☒	☐	Plants

Methods

n/a	Involved in the study	
☒	☐	ChIP-seq
☒	☐	Flow cytometry
☒	☐	MRI-based neuroimaging

Antibodies

Antibodies used	All antibodies were produced in the lab using transient transfection using plasmids expressing the antibody heavy and light chains. Goat-anti-human IgG was obtained from Jackson Immunoresearch (cat#109-035-170) and used at a dilution of 1:3000.
Validation	Antibodies were validated using sequencing. Goat-anti-human IgG was authenticated by manufacturer https://www.jacksonimmuno.com/catalog/products/109-035-170)

Eukaryotic cell lines

Policy information about cell lines and Sex and Gender in Research

Cell line source(s)	HEK293T and HEK293F cell lines were obtained from ATCC or Invitrogen, respectively. Huh-7 were a kind gift from François-Loïc Cosse.
Authentication	HEK293T, and HEK293F were authenticated by the provider (ThermoFisher). Huh-7 were authenticated by provider.
Mycoplasma contamination	All cell lines were tested negative for mycoplasma
Commonly misidentified lines (See ICLAC register)	No commonly misidentiefied cell lines were used

Animals and other research organisms

Policy information about studies involving animals; ARRIVE guidelines recommended for reporting animal research, and Sex and Gender in Research

Laboratory animals	18 rabbits: New Zealand White, female, approximate weight at study time 2.5-3.0kg
Wild animals	The study did not involve wild animals.
Reporting on sex	All animals were female.
Field-collected samples	The study did not involve field-collected samples
Ethics oversight	Animal study was performed by Labcorp (Denver, USA). Study number: 0201-23. All procedures in this study design are in compliance with the U.S. Department of Agriculture's (USDA) Animal Welfare Act (9 CFR Parts 1, 2, and 3); the Guide for the Care and Use of Laboratory Animals (Institute of Laboratory Animal Resources, National Academy Press, Washington, D.C., 2011); and the National Institutes of Health, Office of Laboratory Animal Welfare. Whenever possible, procedures in this study are designed to avoid or minimize discomfort, distress, and pain to animals.

Note that full information on the approval of the study protocol must also be provided in the manuscript.

Plants

Seed stocks	*Report on the source of all seed stocks or other plant material used. If applicable, state the seed stock centre and catalogue number. If plant specimens were collected from the field, describe the collection location, date and sampling procedures.*
Novel plant genotypes	*Describe the methods by which all novel plant genotypes were produced. This includes those generated by transgenic approaches, gene editing, chemical/radiation-based mutagenesis and hybridization. For transgenic lines, describe the transformation method, the number of independent lines analyzed and the generation upon which experiments were performed. For gene-edited lines, describe the editor used, the endogenous sequence targeted for editing, the targeting guide RNA sequence (if applicable) and how the editor was applied.*
Authentication	*Describe any authentication procedures for each seed stock used or novel genotype generated. Describe any experiments used to assess the effect of a mutation and, where applicable, how potential secondary effects (e.g. second site T-DNA insertions, mosiacism, off-target gene editing) were examined.*
