# Supplementary material for: Structure-based design of stable recombinant hepatitis C virus E1E2 heterodimers
Source: Nat Commun. 2026 Jul 23;17:9004. doi: 10.1038/s41467-026-75744-9 (PMC13507286; doi:10.1038/s41467-026-75744-9)
Supplement: Supplementary file 4 [file 41467_2026_75744_MOESM5_ESM.pdf]

nature portfolio

Peer Review File

Structure-based design of stable recombinant hepatitis C virus E1E2 heterodimers

Corresponding Author: Dr Kwinten Sliepen

This file contains all reviewer reports in order by version, followed by all author rebuttals in order by version.

Version 0:

Reviewer comments:

Reviewer #1

(Remarks to the Author)
In this manuscript, Capella-Pujolet al. describe the structure-based design of novel immunogens based on the HCV E1E2 glycoprotein heterodimer. The authors focus particularly on the stabilization of the heterodimer and succeed to produce a stabilized and disulfide-linked heterodimer, which they can produce as soluble immunogen. They design a total of four different heterodimer variants containing a human-derived leucine zipper and one variant omitting it. The authors subsequently characterize these variants regarding a number of parameters. They determine antigenicity and epitope exposure of individual variants using a large antibody panel and use a similar ELISA approach to reveal that antigen stabilization increases thermostability. The authors also demonstrate that antigen stabilization results in higher glycan occupancy, which might contribute to a slightly more desirable antigenicity profile. They also investigate binding of inferred germline precursors of known antibodies, an important parameter for a vaccine candidate, and they show that antigen stabilization slightly enhances binding to a particular type of bnAb precursor antibodies (AR3-specific antibodies). Eventually, the authors determine the structure of a stabilized E1E2 immunogen in complex with a human neutralizing antibody targeting a novel epitope. Importantly, the authors show that their antigen design strategy is not only applicable to a single HCV strain but can be transferred also to other strains and can therefore serve as a general blueprint to stabilize HCV glycoprotein antigens. In spite of the considerable success in antigen stabilization, the authors show that mainly neutralizing antibodies targeting the autologous strain are induced, but only low titers of broadly neutralizing antibodies.
This manuscript provides a very extensive case story, how immunogen design can be used to modify immunogen candidates. The experiments have been carried out carefully and mostly results have been put into the appropriate context. However, addressing a few additional issues could further improve the quality of the presented manuscript.

Major points:
1. The authors consistently use an analogy to the energy landscape of class I fusion proteins, in that they discuss the HCV antigen engineering as stabilization of a “pre-fusion conformation”. For class I fusion proteins the two conformations 1) prior to the fusogenic conformational change and 2) after the fusion event are well-characterized and thus the conformational change has been described in great detail. In contrast, the mechanism of HCV-mediated membrane fusion remains largely elusive and no clear idea has been proposed yet, how a conformational change in the glycoproteins could possibly facilitate merging of the two bilayer membranes. The HCV glycoprotein conformations reported to date are likely describing a conformational state corresponding to a pre-receptor binding and pre-fusion state. Therefore, the use of an analogy like “pre-fusion stabilization” clearly referring to well-characterized viral fusion proteins is misleading the reader and should be removed or at least toned down throughout the manuscript.
2. It is clear from the presented data that C2P2 reduces off target responses against the leucine zipper and improves autologous neutralization against AMS0232. However, the most crucial parameter of a potential HCV immunogen is likely cross-neutralization, and this remains weak for all engineered immunogens according to figure S14. It is a bit surprising to find these results only in a supplementary figure, this should rather be part of the main figure 3 and the text should be modified accordingly. In view of the antigen engineering success (as shown by epitope exposure and thermostability) the poor cross-neutralizing capacity is very surprising – The authors should include a paragraph into the discussion, why these two antigen features seem to be unrelated.
3. Throughout the manuscript the engineered immunogens are presented as superior to existing immunogens, however, it remains elusive, what the reference of the authors for this judgement is. In line 52, they compare it to sE2, but unfortunately, sE2 is later in the manuscript not used as reference anymore, somehow leaving the reader without a clear immunogenicity

benchmark.

Minor points:
1. Was germline binding analysed only for the engineered variants of UKNP4.1.1? As engineered immunogens for other genotypes already exist, it would be straightforward to include those in the germline binding analysis as well. Such an analysis could nicely answer the question, whether the improved germline binding is a general feature of the presented immunogen engineering strategy.
2. It is a bit surprising to this reviewer that the recent report of the HCV glycoprotein complex forming a heterotetramer (Augestad et al., Nature, 2024) is not cited in the introduction at all, although a number of structural aspects are absolutely relevant in this context. This should be appropriately mentioned in the text and cited. Also, in line 82-84 the authors introduce the internal TM region of E1 and how AF2 prediction suggests this motif to buried in the membrane, without mentioning that Augestad et al. showed this membrane insertion. This should be corrected.
3. The glycan analysis is a very informative part of this manuscript. The authors should make sure that their the most relevant engineered immunogen (C2P2) is also included into this analysis.
4. In line 189 the authors describe an "RMSD of 0.856 Å across conserved residues" without specifying, how many conserved residues (or better Cα atoms) they included in this calculation. For clarity and completeness, it would be preferable to report both the RMSD calculated over all Cα atoms and the RMSD calculated over conserved Cα atoms, while explicitly stating the number of conserved Cα atoms used in the analysis.
5. Are the data shown in figure 3A, figure 4B and in figure S12B identical or are these experiments different? Please clarify in the text and the corresponding legends.
6. Why did the authors use the human leucine zipper in the first place, in spite of the known immunogenicity? Please explain.
7. Fig. S2C. The authors state in line 140 that "sE1E2.v2-v4-LZ produced fewer aggregates than sE1E2.v1-LZ". This should be clarified into "a percentage of aggregates slightly lower than 50%" or something similar.
8. Figures 1+3, Figure S2E. Several experiments in the presented manuscript show that introduction of the disulfide bond (v4 vs. v3) does not seem to improve antigenicity but rather decrease epitope exposure. The authors should clarify and address this apparent contradiction briefly in the discussion section.
9. Fig. S14. Color gradients in the tables are not explained and their meaning remains elusive to the reader.

Reviewer #2

(Remarks to the Author)

The paper by Capella-Pujol/Sliepel et al seeks to design recombinant HCV E1E2 heterodimers, to enhance stability of this for use as an HCV antigen.

Through structured design and reiteration, the investigators enhance binding of soluble E1/E2 to conformational Abs and increase the thermal stability of the sE1/E2.
In brief the authours i) replace the hydrophobic putative fusion peptide (pFP) in the HCV E1E2 protein with a short hydrophilic linker that produces a native-like soluble protein, and enhances binding to broadly neutralizing antibodies. ii) Further structure-guided mutations, including proline substitutions and engineered disulfide bonds further increased stability, reduced aggregation, and improved antibody binding and thermal stability creating versions 3 and v4). This approach was applied to several HCV viral strains and enhanced engagement of germline "antibody precursors". They iii) further enhanced antigenicity by creating antigens that lacked the heterodimerisation domain found used in the LZ constructs (C2p2 constructs).
The best construct (C2P2) produces stronger neutralization (~4× higher) than earlier designs to homologous antigen - but responses remain modest were largely strain-specific when assessed in prime boost vaccine regimens in rabbits. There was however minimal evidence of cross- strain neutralisation (shown in Fig. S14E,D)."

Major comments
Overall, the structural based improvements in E1/E2 (the only relevant target of HCV bNAbs) is interesting and the approach taken is rationale, novel, careful and largely comprehensive and cutting edge. There remains a real need for an HCV vaccine and a B cell antigen will be a key component of this. The work is of significant interest in the field. However, the fact that these novel antigens, in spite of all the demonstrated antigenic improvements do not generate bnAbs is an important and key observation, that is not drawn out in the manuscript.

I have the following major comments that would improve the paper.

1. The paper is detailed and carefully written (other than the abstract). However, the crucial fact that although antigenicity is enhanced, these optimised constructs do not generate bNAbs. This crucial data is currently shown only in supplementary figures – and should be bought into the main paper. This is also not mentioned in abstract, and it is not highlighted in the discussion section of the manuscript. Why do the authours think that cross reactive Abs are not generated, despite germline targeting etc. and what should be done about this?

2. No T cell data is shown in the rabbit vaccine experiments – was this measured? If not why not? Do the authors not think a T cell response is an important component of an HCV vaccine?

3. No limitations are given in the manuscript – a paragraph that speaks to this should be included (including all the points

above).

4. Since bNAbs are not generated what are the next steps for these antigens?

5. The authours do not place these findings in the context of other data published in the field – and recent published papers in HCV vaccines are not referenced or referred to (including one recent one in Nature comms)

6. Is it of concern that the v4 construct most closely resembles an neutralisationresistant strain (Figure 3).

7. Why was AT1211 selected for the structural characterisation of the v4 constructs? This is not clear to me. Whilst this interaction is described in great detail, it is not clear how this contributed to vaccine antigen design (lines 182-232)- can this be bought out further?

8. Similarly how would/does higher glycan occupancy contribute to the more favourable antigenic profiled of the V3/V4 constructs?

Minor comments

1. There is no evidence in humans that vaccines that contain the LZ region actually are autoreactive in humans – though I agree this is a theoretical possibility. Please change probably to" possibly" in line 53

2. Figure 3b – is addressing germline Ab binding – this is not clear in the legend

3. The fact that adjuvant (? squalene) is used is not stated in abstract or in the Fig 4 legend – the dose of vaccine given is also omitted here.

4. Page numbering the manuscript would help reviewers (and is missing throughout)

5. Check abbreviations eg pFP are defined (some are not)

6. "Previous E1E2 immunogens" as stated in the abstract is unclear – which previous immunogens? – overall the abstract needs attention. It is also nuclear in the abstract that "heterodimerisation domains" refer to the LZ domains of Guest et al – assuming it does.

Reviewer #3

(Remarks to the Author)
The study explores an important and cutting-edge approach to HCV B-cell vaccine design, namely how to best solubilize and stabilize E1E2 heterodimers. It will be important to ascertain whether this approach, which can naturally incorporate epitopes from epitope clusters like AR4 and AR5 (not present on E2 based antigens) is, in fact, a superior alternative to E2 based antigens.
With a few minor caveats, the authors do a wonderful job of describing the rationale for their study as well as explaining the process by which they design their experiments and use them to inform the next. It is a rare pleasure to read a paper, dealing with protein structures, including complicated protein designs, and being able to follow the flow of the manuscript in the first read-through. The results regarding E1E2 solubilization are only partly shown before by He et al, 2026, where the pFP was similarly removed, but none of the stabilizing mutations, nor the cys-cys bridge wee described before. The authors are correct that these findings will be beneficial to other researchers doing similar projects to develop a much-needed HCV vaccine. I really only have one major criticism (see below):

Major point:
1. The subject of the study is highly pertinent and HCV researchers worldwide are excited to learn how well E1E2 heterodimers can be made to work. The main point of criticism is therefore also leveled at the minimal immunization studies performed here. Specifically that E2 designs were not included for comparison and that VLPs were not used to potentially boost heterologous responses. This 2nd point becomes salient particularly because only autologous neutralization was observed. In fact, it seems likely from several other virus (as well as HCV) studies, including the recent study by He et al, that testing these E1E2 heterodimer designs on VLPs, might have resulted in boosting the heterologous response. It seems strange, given the experience of the authors, that this was not attempted, particularly since the lack of cross-neutralization is a significant shortcoming of the E1E2 approach as presented here.

Minor points:
1. The authors perform several analyses of the pFP, arriving at the conclusion that it is possibly a transmembrane region itself. However, they fail to mention perhaps the strongest support for this, which is the paper published in 2024 by Augestad and Olesen et al. (though it is cited later in the Discussion), in which the complete TM helix bundle is shown to include the

pFP alpha helix. In fact, while it is indeed possible that a future successful HCV vaccine candidate will be some variation of soluble E1E2 heterodimers, it seems an odd omission to not mention in the introduction that these E1E2 heterodimers themselves seem to assemble into homodimeric arrangements as this does not, by itself, invalidate the approach of trying to use E1E2 heterodimers.

2. Fig 1. The “D” is on top of the panel.

3. In Fig 4, it would be helpful to mention in the legend or on the y axis that the neutralization data is “Autologous neutralization”

Version 1:

Reviewer comments:

Reviewer #1

(Remarks to the Author)
The authors addressed the raised concerns.

Reviewer #2

(Remarks to the Author)
Thank you for the response to my review

1. Please amend the abstract to say responses to homologous but not heterologous Abs were generated (autologous is not the correct term) - and as said the fact that no heterologous responses were generated should be displayed in the abstract. Figure 4H is welcome addition to the paper

2. There is still no paragraph that addressed study limitations in the discussion.

3. Whilst I acknowledge that this antigen is a B cell antigen - T cell responses - and especially CD4+ T helper responses - to this same antigen are important in generating long lived/ SHM B cells etc. The fact that these have not been measured in any animal model shoud be added as a limitation to the paper (amongst others mentioned already).

Open Access This Peer Review File is licensed under a Creative Commons Attribution 4.0 International License, which permits use, sharing, adaptation, distribution and reproduction in any medium or format, as long as you give appropriate credit to the original author(s) and the source, provide a link to the Creative Commons license, and indicate if changes were

made.
In cases where reviewers are anonymous, credit should be given to 'Anonymous Referee' and the source.
The images or other third party material in this Peer Review File are included in the article's Creative Commons license, unless indicated otherwise in a credit line to the material. If material is not included in the article's Creative Commons license and your intended use is not permitted by statutory regulation or exceeds the permitted use, you will need to obtain permission directly from the copyright holder.
To view a copy of this license, visit https://creativecommons.org/licenses/by/4.0/

REVIEWER COMMENTS

Reviewer #1 (Remarks to the Author):

In this manuscript, Capella-Pujol et al. describe the structure-based design of novel immunogens based on the HCV E1E2 glycoprotein heterodimer. The authors focus particularly on the stabilization of the heterodimer and succeed to produce a stabilized and disulfide-linked heterodimer, which they can produce as soluble immunogen. They design a total of four different heterodimer variants containing a human-derived leucine zipper and one variant omitting it. The authors subsequently characterize these variants regarding a number of parameters. They determine antigenicity and epitope exposure of individual variants using a large antibody panel and use a similar ELISA approach to reveal that antigen stabilization increases thermostability. The authors also demonstrate that antigen stabilization results in higher glycan occupancy, which might contribute to a slightly more desirable antigenicity profile. They also investigate binding of inferred germline precursors of known antibodies, an important parameter for a vaccine candidate, and they show that antigen stabilization slightly enhances binding to a particular type of bnAb precursor antibodies (AR3-specific antibodies). Eventually, the authors determine the structure of a stabilized E1E2 immunogen in complex with a human neutralizing antibody targeting a novel epitope. Importantly, the authors show that their antigen design strategy is not only applicable to a single HCV strain but can be transferred also to other strains and can therefore serve as a general blueprint to stabilize HCV glycoprotein antigens. In spite of the considerable success in antigen stabilization, the authors show that mainly neutralizing antibodies targeting the autologous strain are induced, but only low titers of broadly neutralizing antibodies.
This manuscript provides a very extensive case story, how immunogen design can be used to modify immunogen candidates. The experiments have been carried out carefully and mostly results have been put into the appropriate context. However, addressing a few additional issues could further improve the quality of the presented manuscript.

We thank the reviewer for highlighting the breadth of the presented work and the insightful comments, to which we have responded in a point-by-point basis below.

Major points:

1. The authors consistently use an analogy to the energy landscape of class I fusion proteins, in that they discuss the HCV antigen engineering as stabilization of a "pre-fusion conformation". For class I fusion proteins the two conformations 1) prior to the fusogenic conformational change and 2) after the fusion event are well-characterized and thus the conformational change has been described in great detail. In contrast, the mechanism of HCV-mediated membrane fusion remains largely elusive and no clear idea has been proposed yet, how a conformational change in the glycoproteins could possibly facilitate merging of the two bilayer membranes. The HCV glycoprotein conformations reported to date are likely describing a conformational state corresponding to a pre-receptor binding and pre-fusion state. Therefore, the use of an analogy like "pre-fusion stabilization" clearly referring to well-characterized viral fusion proteins is misleading the reader and should be removed or at least toned down throughout the manuscript.

The reviewer brings up a valid point. The mechanism of entry of E1E2 and the conformational changes that happen during membrane fusion have yet to be elucidated. However, recent studies, either (prediction-based) structural work (Augestad and Holmboe Olesen et al. *Nature* 2024; Oliver et al. *PLoS Biol* 2023; Kumar et al. *Nature* 2021) or modelling of the viral entry mechanism using mutagenesis (Pfaff-Kilgore et al. *Cell Rep* 2022) suggest E1E2 exists in different states, which probably includes pre-fusion (a broad term that we surmise also encompasses the pre-receptor binding state) and post-fusion states (i.e. after membrane fusion).

Previously, we have shown that the AR4A bNAb targets an epitope on E2 outside of the CD81 receptor binding site (Torrents et al. *Science* 2022). Furthermore, E2 alone does not bind AR4A and E1 is required for binding (Giang et al. PNAS 2012). Since AR4A neutralizes HCV and blocks viral entry, this implies AR4A locks E1E2 in a "pre-fusion" state. The W326P and L682P mutations enhance AR4A (and AT1618) binding are akin to proline mutations aimed at stabilization of the pre-fusion state of class I (i.e. HIV-1 Env SOSIP, SARS-CoV-2 spike and numerous others) and class III glycoproteins (Rhabies G: Ng et al. *CHM* 2022). Lastly, the antigenicity profiles of our designs more closely mimic the antigenicity of infectious E1E2 on HCV pseudoparticles, which per definition should resemble pre-fusion E1E2 (Fig 1D, Fig S3B). Together, we deemed it appropriate to refer to this as pre-fusion stabilization. On the other hand, we agree that a lack of structural information on the states of E1E2 prevents definitive statements and we have therefore toned down the manuscript throughout or added additional context (lines 43-52) of the introduction) and referred to our constructs as being more "native-like" rather than pre-fusion form wherever appropriate. However, we believe that the analogy with the work done for other viral fusion class proteins is valid and we decided to clarify the original discussion section (lines 428-435), to help the reader throughout the story to understand our reasoning.

2. It is clear from the presented data that C2P2 reduces off target responses against the leucine zipper and improves autologous neutralization against AMS0232. However, the most crucial parameter of a potential HCV immunogen is likely cross-neutralization, and this remains weak for all engineered immunogens according to figure S14. It is a bit surprising to

find these results only in a supplementary figure, this should rather be part of the main figure 3 and the text should be modified accordingly. In view of the antigen engineering success (as shown by epitope exposure and thermostability) the poor cross-neutralizing capacity is very surprising – The authors should include a paragraph into the discussion, why these two antigen features seem to be unrelated.

Reviewers #2 and #3 bring up a similar point, and we should have been clearer about the goals of this immunogenicity study. First, our main goal was to generate a design that produce a stable native-like soluble form of E1E2. We next showed it can be used to generate E1E2 from other strains and finally a soluble native-like E1E2 design that does not require scaffolds. The rabbit immunization study was a pilot to see the effects of the stabilizing mutations and scaffold removal on immunogenicity. Like what we observed for HIV-1 Env (del-Moral-Sanchez et al. *npj Vaccines* 2022), we found that stabilization of E1E2 alone is not enough to sufficiently broaden the immune response, although autologous responses seemed to be enhanced (i.e. when Jun/Fos was removed). We are currently applying our template to more immunogenic E1E2 sequences and applying them to broaden the response (i.e. using cocktails, nanoparticle display, germline-targeting), which we deemed outside of the scope of the current study. Additionally, the designs also provide the possibility to produce soluble E1E2 for a wide range of applications: we have also used our proteins to study bispecifc antibody interactions (Radic et al. PNAS 2025) (now added line 458-459). We have adapted the text (added line 366, edited lines 398-402, added lines 504-510) and added Figure 4H.

3. Throughout the manuscript the engineered immunogens are presented as superior to existing immunogens, however, it remains elusive, what the reference of the authors for this judgement is. In line 52, they compare it to sE2, but unfortunately, sE2 is later in the manuscript not used as reference anymore, somehow leaving the reader without a clear immnogenicity benchmark.

We understand the confusion from line 52 and agree we did not phrase it properly. The starting point of our work is based on the design by Guest and colleagues (referred to as sE1E2.v1-LZ). They already performed immunogenicity studies and compared directly with soluble E2 (Wang et al. *PNAS* 2022). Furthermore, sE2 alone does not bind AR4A and our goal was to measure the effect of the stabilizing mutations and scaffold removal on immunogenicity of E1E2, and not to compare E1E2 to E2. Lastly, we would have needed an additional group of rabbits and we preferred to use the limited number of animals at our disposal to explore strategies for broadening the response, which are part of a follow-up study. Therefore, we decided to not include sE2 in our study. We hope that the changes in the text in lines 60 (old line 52) in the introduction and lines 366 in the results section will satisfy the reviewer.

Minor points:

1. Was germline binding analysed only for the engineered variants of UKNP4.1.1? As engineered immunogens for other genotypes already exist, it would be straightforward to include those in the germline binding analysis as well. Such an analysis could nicely answer the question, whether the improved germline binding is a general feature of the presented immunogen engineering strategy.

We used UKNP4.1.1 strain as we previously observed this strain engages several VH1-69-derived inferred germline bNAbs (Capella-Pujol et al. *Nat. Comm*. 2023). Since we found that our designs enhanced mature bNAb binding, our goal here was to determine if this improved folding would also benefit the capacity of UKNP4.1.1 E1E2 to bind inferred germline bNAbs. Our goal was not to find better germline-targeting candidates, which is the topic of a separate follow-up study. We can indicate that optimized sequences are necessary to improve inferred germline bNAb binding (Mulder, Cannac, Capella-Pujol et al. *manuscript in preparation*) and stabilizing mutations alone are not enough to enable inferred germline bNAb binding. We have now adjusted the text slightly (lines 320-322).

2. It is a bit surprising to this reviewer that the recent report of the HCV glycoprotein complex forming a heterotetramer (Augestad et al., Nature, 2024) is not cited in the introduction at all, although a number of structural aspects are absolutely relevant in this context. This should be appropriately mentioned in the text and cited. Also, in line 82-84 the authors introduce the internal TM region of E1 and how AF2 prediction suggests this motif to buried in the membrane, without mentioning that Augestad et al. showed this membrane insertion. This should be corrected.

The reviewer brings up an important paper and a similar points is also brought up by reviewers #2 and #3. We would like to emphasize that we started our design campaign before the homodimer paper was published: our v2 design was based on the insights that the Alphafold predictions provided. We now added additional text in the results to contextualize our initial Alphafold findings with that of homodimer structure (lines 94-96) and now mention the homodimer structure in the introduction (lines 29).

3. The glycan analysis is a very informative part of this manuscript. The authors should make sure that their the most relevant engineered immunogen (C2P2) is also included into this analysis.

This data has now been added. It is now discussed in the results text (lines 360-361) and the detailed glycosylation is part of supplementary figure 14.

4. In line 189 the authors describe an “RMSD of 0.856 Å across conserved residues” without specifying, how many conserved residues (or better Cα atoms) they included in this calculation. For clarity and completeness, it would be preferable to report both the RMSD calculated over all Cα atoms and the RMSD calculated over conserved Cα atoms, while explicitly stating the number of conserved Cα atoms used in the analysis.

We thank the reviewers for the insight. We have edited the text to provide updated RMSD (lines 201-204), on Cα atoms, on the specific number of residues. We removed the word “conserved” as it was misleading, as all (built) residues are identical between the models.

5. Are the data shown in figure 3A, figure 4B and in figure S12B identical or are these experiments different? Please clarify in the text and the corresponding legends.

The data in figure 3A and S12B come from the same experiment. In figure 3A the data is compiled to summarize detailed data shown in figure S12B. Figure 4B contains data from Figure 1B and is used as comparison. We have adapted the figure captions to clarify this.

6. Why did the authors use the human leucine zipper in the first place, in spite of the known immunogenicity? Please explain.

When we initiated our design campaign, the only soluble sE1E2 heterodimer available at the time that bound AR4A, which is a proxy for native-like folding, showed the human LZ domain was necessary for its folding (Guest et al. *PNAS* 2021). Our initial goal was to improve antigenicity of that design using stabilizing mutations. We figured that adding another variable (e.g. by using another dimerization domain) might convolute our interpretations, since we already used another strain than Guest et al. (AMS0232 and not H77c), because we based our designed mutations on the cryo-EM structure of AMS0232 E1E2 (Torrents et al. *Science* 2022). The LZ-based design simultaneously provided a template and benchmark to test our mutations in, and we decided to replace it once we had a stable design. This seemed to have been the right approach, because the resulting designs are compatible with the SYNZIP dimerization domain (Supplemental Fig 13) and it finally allowed us to remove dimerization domains altogether (Figure 4).

7. Fig. S2C. The authors state in line 140 that “sE1E2.v2-v4-LZ produced fewer aggregates than sE1E2.v1-LZ”. This should be clarified into “a percentage of aggregates slightly lower than 50%” or something similar.

We have adapted the text to be more precise in our statements (lines 150-153).

8. Figures 1+3, Figure S2E. Several experiments in the presented manuscript show that introduction of the disulfide bond (v4 vs. v3) does not seem to improve antigenicity but rather decrease epitope exposure. The authors should clarify and address this apparent contradiction briefly in the discussion section.

Indeed, at first sight, it seems that v3 is superior to v4 in terms of antigenicity (Fig 1B). However, v4 is more native-like as it shows a better correlation between the binding and neutralization as shown per figure 1D and figure S3. A testament to the latter, is that introduction of the disulfide bond decreased binding to non-Nabs CBH-4B, CBH-4D and/or CBH-4G (Figures 1B, S2, S12B). We have added the text in lines 194 in the results and lines 454-459 to further clarify these points.

9. Fig. S14. Color gradients in the tables are not explained and their meaning remains elusive to the reader.

We appreciate the reviewer for bringing this up and the figure caption has been adapted.

Reviewer #2 (Remarks to the Author):

The paper by Capella-Pujol/Sliepen et al seeks to design recombinant HCV E1E2 heterodimers, to enhance stability of this for use as an HCV antigen.

Through structured design and reiteration, the investigators enhance binding of soluble E1/E2 to conformational Abs and increase the thermal stability of the sE1/E2.
In brief the authours i) replace the hydrophobic putative fusion peptide (pFP) in the HCV E1E2 protein with a short hydrophilic linker that produces a native-like soluble protein, and enhances binding to broadly neutralizing antibodies. ii) Further structure-guided mutations, including proline substitutions and engineered disulfide bonds further increased stability, reduced aggregation, and improved antibody binding and thermal stability creating versions 3 and v4). This approach was applied to several HCV viral strains and enhanced engagement of germline "antibody precursors". They iii) further enhanced antigenicity by creating antigens that lacked the heterodimerisation domain found used in the LZ constructs (C2p2 constructs).
The best construct (C2P2) produces stronger neutralization (~4× higher) than earlier designs to homologous antigen - but responses remain modest were largely strain-specific when assessed in prime boost vaccine regimens in rabbits. There was however minimal evidence of cross- strain neutralisation (shown in Fig. S14E,D)."

<u>**Major comments**</u>:

Overall, the structural based improvements in E1/E2 (the only relevant target of HCV bNAbs) is interesting and the approach taken is rationale, novel, careful and largely comprehensive and cutting edge. There remains a real need for an HCV vaccine and a B cell antigen will be a key component of this. The work is of significant interest in the field. However, the fact that these novel antigens, in spite of all the demonstrated antigenic improvements do not generate bnAbs is an important and key observation, that is not drawn out in the manuscript.

We thank the reviewer for recognizing the novelty of our study and appreciation of our design approach. We have addressed the comments point by point and we hope the reviewer is pleased with our answers and modifications.

I have the following major comments that would improve the paper.

1. The paper is detailed and carefully written (other than the abstract). However, the crucial fact that although antigenicity is enhanced, these optimised constructs do not generate bNAbs. This crucial data is currently shown only in supplementary figures – and should be brought into the main paper. This is also not mentioned in abstract, and it is not highlighted in the discussion section of the manuscript. Why do the authours think that cross reactive Abs are not generated, despite germline targeting etc. and what should be done about this? It is important to note that (improved) binding to bNAbs does not automatically result in the induction of bNAbs, i.e. antigenicity does not equate immunogenicity (Sanders et al. *PloS Pathogens* 2013, Sanders et al. *Science* 2015, del-Moral-Sanchez et al. npj *Vaccines* 2022): it is an indication that the antigen is well-folded and might have the capacity to induce similar mAbs. However, for sequence-diverse viruses such as HCV, induction of cross-reactive neutralizing antibodies will probably require optimized vaccine strategies, such as cocktail vaccines, mRNA vaccines, (mosaic) nanoparticles (Sliepen et al. *Nat Comm*. 2022, He et al. *Nat Comm* 2026) and/or generating germline-targeting native-like E1E2 immunogens. The designs described here can be used to generate such immunogens. We are currently exploring such antigen designs and strategies, and we will present these data in other manuscripts. Furthermore, rabbits and wild-type mice are not suitable to explore HCV germline-targeting since these animals do not express orthologues of the human VH1-69 B cell receptor gene. Immunizing animals carrying (orthologues of) VH1-69 would be more appropriate to test germline-targeting strategies.

The goal of the immunogenicity study in Figure 4 was to test the effect of the stabilizing mutations and removal of the leucine zipper (LZ). We observed redirection of the immune response towards E1E2 when the leucine zipper was removed, but this did not result in a broader neutralizing response. This is akin to how masking of immunodominant epitopes on HIV-1 Env can redirect responses to subdominant epitopes, but this does not necessarily result in the induction of bNAbs (del Moral-Sanchez et al. *npj Vaccines* 2022).

We have now changed the text and, because of a similar comment of reviewer #1 (major point 2) and #3 (major point 1), where we acknowledge that this point is relevant and added figure 4H and added line 366, edited lines 398-402, added lines 504-510 to the main text.

2. No T cell data is shown in the rabbit vaccine experiments – was this measured? If not why not? Do the authors not think a T cell response is an important component of an HCV vaccine?

While we think T cells will be an important component of an HCV vaccine, the focus of this work is on a B cell vaccine antigen. Therefore, we did not measure T cell response in this study, and we usually do not measure such responses in rabbits (Sliepen et al. *Nat Comm*. 2022, del-Moral-Sanchez et al. *npj Vaccines* 2022, Sanders et al. *Science* 2015). Furthermore, we do not expect (major) differences in T cell responses between differentially stabilized antigens, while it would have required additional costs and time (shipping, but also assay setup for measuring rabbit T cell responses). Again, similar to point 1, this was a pilot study of a B cell vaccine, not that of a perfect B cell vaccine, let alone a perfect HCV vaccine. We

have raised the point of the importance of T cells in the discussion for future work (lines 474).

3. No limitations are given in the manuscript – a paragraph that speaks to this should be included (including all the points above).

We thank the reviewer for bringing this up. We have modified the text in the discussion to further comment on the points above (as well as for other reviewers) in lines 467-470, 474, but also that our designs are probably compatible with the E1E2 homodimer arrangement (line 512-515).

4. Since bNAbs are not generated what are the next steps for these antigens?

See our response to major point 1: we have now included in the text our intentions for future and ongoing work on how to use our stabilization strategy to design novel antigens that can generate a broad response against HCV (lines 474, 504-510, 512-515).

5. The authours do not place these findings in the context of other data published in the field – and recent published papers in HCV vaccines are not referenced or referred to (including one recent one in Nature comms)

Our work is mainly focused on the stabilization of soluble secreted E1E2 for others to generate novel immunogens or native-like antigens for a broad spectrum of use cases. Furthermore, the paper that the reviewer brings up was referenced in our discussion when it was still a preprint (He et al. bioRxiv) and was published in Nature Communications while this paper was being under review. We have added some text and the reference in the results section (lines 123-124), and hope that we contextualized better our results with other HCV vaccine studies and added a reference in the discussion that showcases how we used our stabilized E1E2 to study bispecific antibody binding (see line 459) (Radić et al. *PNAS* 2025).

6. Is it of concern that the v4 construct most closely resembles a neutralisation resistant strain (Figure 3).

To the contrary, we do not think that sE1E2.v4-LZ resembles a neutralization resistant strain persé, but rather more closely resembles the E1E2 heterodimer on the virus: see also our response to minor point 8 of reviewer 1. The sE1E2.v4-LZ is the most native-like as its antigenicity more strongly correlated with neutralization (Fig. 1D and S3B) and showed low non-NAb binding (Fig 1B). The fact of more “native-like-ness” of the sE1E2.v4 design was confirmed by the antigenicity profile of AMS0230 sE1E2.v4-LZ (lines 290-294), which is based on the unusually neutralization-resistant AMS0230 strain (Chumbe et al. *Virus Research* 2024), but on the other hand, H77 E1E2.v4-LZ showcased an antigenicity profile that resembles a more neutralization-sensitive strain, as expected (Fig 3A, Fig S12). As such, the sE1E2.v4-LZ can be used to generate antigenic mimics of neutralization-sensitive and neutralization-resistant strains alike. We also added lines 508-510.

7. Why was AT1211 selected for the structural characterisation of the v4 constructs? This is not clear to me. Whilst this interaction is described in great detail, it is not clear how this contributed to vaccine antigen design (lines 182-232)- can this be bought out further? Thus far, the AT1211 epitope was not yet resolved. It was not specifically used to design the constructs as much as it was to use the opportunity provided by our stabilized designs to solve a structure of a novel epitope. We have now added a small sentence to clarify this reasoning (lines 197-198).

8. Similarly how would/does higher glycan occupancy contribute to the more favourable antigenic profiled of the V3/V4 constructs?

We thank the reviewer for pointing out that this was not clear in the text. First, since we aimed to generate a native-like antigen, we were satisfied to observe that the v3 and v4 glycan occupancy is higher than that of v1 and therefore more closely resembles the glycan occupancy of membrane-bound E1E2 (Torrents et al. *Science* 2022). Second, removing glycans affect infectivity and neutralization sensitivity (Prentoe et al. *PNAS* 2019). Additionally, we showed that glycan on position 623 interacts with the light chain of AR4A (Torrents et al. *Science* 2022) and since 623 glycosylation is increased in v3 and v4, this might also partially explain their improved binding to AR4A. We have expanded our results and discussion to try and give a better explanation about how glycans contribute to better antigenic profile on the V3/V4 (lines 271-275) and added a reference in line 454.

Minor comments:

1. There is no evidence in humans that vaccines that contain the LZ region actually are autoreactive in humans – though I agree this is a theoretical possibility. Please change probably to" possibly" in line 53

Agree, and we have now changed the text to "possibly".

2. Figure 3b – is addressing germline Ab binding – this is not clear in the legend We have adapted the figure and its caption to make it clear it is germline antibody binding.

3. The fact that adjuvant (? squalene) is used is not stated in abstract or in the Fig 4 legend – the dose of vaccine given is also omitted here.

We have included the dosage and the adjuvant in Fig 4D and added "adjuvanted" in the abstract.

4. Page numbering the manuscript would help reviewers (and is missing throughout)

We have added page numbering to the manuscript.

5. Check abbreviations eg pFP are defined (some are not)

We thank the reviewer for pointing this out and have checked accordingly.

6. "Previous E1E2 immunogens" as stated in the abstract is unclear – which previous immunogens? – overall the abstract needs attention. It is also unclear in the abstract that "heterodimerisation domains" refer to the LZ domains of Guest et al – assuming it does.

We thank the reviewer for pointing this out. We have adapted the abstract and hope it is clear now.

Reviewer #3 (Remarks to the Author):

The study explores an important and cutting-edge approach to HCV B-cell vaccine design, namely how to best solubilize and stabilize E1E2 heterodimers. It will be important to ascertain whether this approach, which can naturally incorporate epitopes from epitope clusters like AR4 and AR5 (not present on E2 based antigens) is, in fact, a superior alternative to E2 based antigens.

With a few minor caveats, the authors do a wonderful job of describing the rationale for their study as well as explaining the process by which they design their experiments and use them to inform the next. It is a rare pleasure to read a paper, dealing with protein structures, including complicated protein designs, and being able to follow the flow of the manuscript in the first read-through. The results regarding E1E2 solubilization are only partly shown before by He et al, 2026, where the pFP was similarly removed, but none of the stabilizing mutations, nor the cys-cys bridge were described before. The authors are correct that these findings will be beneficial to other researchers doing similar projects to develop a much-needed HCV vaccine. I really only have one major criticism (see below):

We appreciate the positive comments of the reviewer on our work and we thank the reviewer for appreciating the flow of the manuscript as we spend a lot of time and effort in writing this manuscript. Below, we have detailed reply to the points brought up.

Major point:

1. The subject of the study is highly pertinent and HCV researchers worldwide are excited to learn how well E1E2 heterodimers can be made to work. The main point of criticism is therefore also leveled at the minimal immunization studies performed here. Specifically that E2 designs were not included for comparison and that VLPs were not used to potentially boost heterologous responses. This 2nd point becomes salient particularly because only autologous neutralization was observed. In fact, it seems likely from several other virus (as well as HCV) studies, including the recent study by He et al, that testing these E1E2 heterodimer designs on VLPs, might have resulted in boosting the heterologous response. It seems strange, given the experience of the authors, that this was not attempted, particularly since the lack of cross-neutralization is a significant shortcoming of the E1E2 approach as presented here.

Like reviewers #1 and #2, this reviewer brings up the limited capacity of our designs to generate a strong and broad neutralizing response in rabbits. As we mentioned earlier (reviewer #1, major comment 3), we did not use sE2, because 1) sE1E2.v1-LZ was previously compared to sE2 (Wang et al. *PNAS* 2022) 2) we wanted to understand the effect of the mutations on immunogenicity and 3) we would have needed an additional group of rabbits. We have addressed the limited cross-neutralizing responses in our responses to reviewer #1 (major comment 2) and reviewer #2 (major comment 1). In short, stabilizing E1E2 in a native-like monomeric form is not enough for decent cross-neutralization. Currently, we are exploring other strategies to improve immunogenicity and given our experience, this includes nanoparticles for the multivalent presentation of E1E2. We have now added Figure 4H on the cross-neutralization and and added line 366, edited lines 398-402, added lines 504-510 to the main text, also to highlight potential future work.

<u>Minor points:</u>

1. The authors perform several analyses of the pFP, arriving at the conclusion that it is possibly a transmembrane region itself. However, they fail to mention perhaps the strongest support for this, which is the paper published in 2024 by Augestad and Olesen et al. (though it is cited later in the Discussion), in which the complete TM helix bundle is shown to include the pFP alpha helix. In fact, while it is indeed possible that a future successful HCV vaccine candidate will be some variation of soluble E1E2 heterodimers, it seems an odd omission to not mention in the introduction that these E1E2 heterodimers themselves seem to assemble into homodimeric arrangements as this does not, by itself, invalidate the approach of trying to use E1E2 heterodimers.

As stated in our response to reviewer #1 (minor point #2), we initially started our design campaign before the Augestad paper was published and based our designs on the described Alphafold predictions. We have now added lines 94-96 in the results to contextualize our design in light of the homodimer structure and in the discussion added lines 512-515 to highlight that our stabilizing mutations should also be compatible with the homodimeric arrangement of E1E2.

2. Fig 1. The “D” is on top of the panel.

We thank the reviewer for pointing this out and have changed the figure.

3. In Fig 4, it would be helpful to mention in the legend or on the y axis that the neutralization data is “Autologous neutralization

The figure has been adapted.

Reviewer #1 (Remarks to the Author):

The authors addressed the raised concerns.

Reviewer #2 (Remarks to the Author):

Thank you for the response to my review

1. Please amend the abstract to say responses to homologous but not heterologous Abs were generated (autologous is not the correct term) - and as said the fact that no heterologous responses were generated should be displayed in the abstract. Figure 4H is welcome addition to the paper

We have now adjusted the abstract and happy to hear the addition of Figure 4H is appreciated.

2. There is still no paragraph that addressed study limitations in the discussion.
A “Limitations of this study” (akin to articles in Cell portfolio journals) is not part of the style of the Nature portfolio journals. We previously noted the limited cross-neutralization as a limitation before and have now adjusted the text to make this point more explicit (line 469-471). We have now also added a section regarding the lack of measurement of T cell responses and highlighted that rabbits are maybe the most ideal animal model for measuring antibody responses against HCV (lines 479-485).

3. Whilst I acknowledge that this antigen is a B cell antigen - T cell responses - and especially CD4+ T helper responses - to this same antigen are important in generating long lived/ SHM B cells etc. The fact that these have not been measured in any animal model shoud be added as a limitation to the paper (amongst others mentioned already).

See point 2: we have now added a sentence that acknowledges that the lack of T cell response measurements is a limitation of this study.
